# Age-Related Differences in Test-Retest Reliability in Resting-State Brain Functional Connectivity

**DOI:** 10.1371/journal.pone.0049847

**Published:** 2012-12-05

**Authors:** Jie Song, Alok S. Desphande, Timothy B. Meier, Dana L. Tudorascu, Svyatoslav Vergun, Veena A. Nair, Bharat B. Biswal, Mary E. Meyerand, Rasmus M. Birn, Pierre Bellec, Vivek Prabhakaran

**Affiliations:** 1 Department of Biomedical Engineering, University of Wisconsin-Madison, Madison, Wisconsin, United States of America; 2 Department of Elec. and Comp. Engineering, University of Wisconsin-Madison, Madison, Wisconsin, United States of America; 3 Neuroscience Training Program, University of Wisconsin-Madison, Madison, Wisconsin, United States of America; 4 Department of Medicine, University of Pittsburgh, Pittsburgh, Pennsylvania, United States of America; 5 Department of Medical Physics, University of Wisconsin-Madison, Madison, Wisconsin, United States of America; 6 Department of Radiology, University of Wisconsin-Madison, Madison, Wisconsin, United States of America; 7 Department of Radiology, University of Medicine and Dentistry of New Jersey, Newark, New Jersey, United States of America; 8 Department of Psychiatry, University of Wisconsin-Madison, Madison, Wisconsin, United States of America; 9 Geriatric Institute Research Center, Universite de Montreal, Montreal, Quebec, Canada; Yale University, United States of America

## Abstract

Resting-state functional MRI (rs-fMRI) has emerged as a powerful tool for investigating brain functional connectivity (FC). Research in recent years has focused on assessing the reliability of FC across younger subjects within and between scan-sessions. Test-retest reliability in resting-state functional connectivity (RSFC) has not yet been examined in older adults. In this study, we investigated age-related differences in reliability and stability of RSFC across scans. In addition, we examined how global signal regression (GSR) affects RSFC reliability and stability. Three separate resting-state scans from 29 younger adults (18–35 yrs) and 26 older adults (55–85 yrs) were obtained from the International Consortium for Brain Mapping (ICBM) dataset made publically available as part of the 1000 Functional Connectomes project www.nitrc.org/projects/fcon_1000. 92 regions of interest (ROIs) with 5 cubic mm radius, derived from the default, cingulo-opercular, fronto-parietal and sensorimotor networks, were previously defined based on a recent study. Mean time series were extracted from each of the 92 ROIs from each scan and three matrices of *z*-transformed correlation coefficients were created for each subject, which were then used for evaluation of multi-scan reliability and stability. The young group showed higher reliability of RSFC than the old group with GSR (*p*-value = 0.028) and without GSR (*p*-value <0.001). Both groups showed a high degree of multi-scan stability of RSFC and no significant differences were found between groups. By comparing the test-retest reliability of RSFC with and without GSR across scans, we found significantly higher proportion of reliable connections in both groups without GSR, but decreased stability. Our results suggest that aging is associated with reduced reliability of RSFC which itself is highly stable within-subject across scans for both groups, and that GSR reduces the overall reliability but increases the stability in both age groups and could potentially alter group differences of RSFC.

## Introduction

Since the discovery that the human brain at rest consists of spatially distributed but functionally connected regions in which coherent patterns of low-frequency fluctuations in the blood oxygen level-dependent (BOLD) signal are temporally correlated [1], resting-state functional MRI (rs-fMRI) has been used extensively for investigating brain functional connectivity (FC). Resting-state functional connectivity (RSFC) provides insight into the large scale structure of interactions between brain regions that support the integrated deviations of disease states from normally observed human health. These deviations may be a fundamental causative factor in both neuropathologic and neurodevelopmental conditions such as dementia [2]–[3], autism [4]–[6], schizophrenia [7]–[9], depression [10]–[11] and other conditions.

The reliability and stability of the RSFC method is critical to establish in normals as well as in normal development and normal aging so that deviations from these healthy states can be assigned to a particular disease state with certainty. Here, reliability is defined as the reproducibility of functional connections for a given subject across scan sessions, quantified with Intraclass Correlation coefficients (ICC). Reliability of RSFC has been evaluated in healthy young adults [12], and in healthy children as well as adolescents [13]. Stability is defined as the consistency of functional connections for a given subject across scans (within-subject) or in a given scan session across subjects (between-subject), quantified with Kendall’s coefficient of concordance (*W*). Spatial consistency of RSFC has been demonstrated on young normal adults [14]–[16]. Although these results are encouraging, much more work needs to be done in order to explore normal age-related differences in RSFC. Previous studies on functional brain networks indicated that cost efficiency was reduced significantly in older normal people based on analyses of network efficiency [17], and that normal aging was associated with changes in modular organization of functional networks based on a graph theoretical analysis [18]. Furthermore, the modular organization of structural brain networks was similar between the young and middle age groups, but quite different from the old group based on an analysis of topological organization of structural brain networks in healthy individuals [19]. Another recent study found significant group-level variance in FC maps between young and old groups [20]. However, to our knowledge, test-retest reliability and stability in RSFC has not yet been explicitly quantified in older adults. The main purpose of this study is to investigate test-retest reliability and stability of the RSFC parameters in both young and old groups.

In the present study, we divided all healthy subjects into two extreme groups by age. Study participants were selected from the ICBM dataset. The functional connectivity in the human brain consisting of 92 regions (**Figure S1**) was constructed by computing the correlation matrices across subjects and across each of the three scans. Similar to the method demonstrated in a recent study by Shehzad et al. [12] in which they assessed intersession (between-scan time-interval >5 months), intrasession (between-scan time interval <1 hour), and multi-scan (across all 3 scans) reliability and stability on 26 young adults, we measured multi-scan reliability and stability on both young and old adults based on region-of-interest (ROI) analyses. Our first goal was to compare test-retest reliability and stability of RSFC across scans between the young and old group.

Global signal, the spatial average of local signals from all cerebral voxels, has been suggested as a nuisance regressor for artifact reduction as it reflects coherent signal fluctuations across the brain [21]. While some studies indicate improved fMRI results after global signal regression [22], others suggest to avoid global scaling in fMRI analysis as it may decrease statistical power [23] and cause anti-correlations [24]. Our second goal in this study was to investigate how global signal regression (GSR) affects RSFC and the test-retest reliability and stability.

## Results

### Reliability of Functional Connectivity

To investigate the between-group differences of RSFC reliability, we calculated multi-scan ICCs for each correlation across all 3 scans for each group (**Table 1**). A reasonable criterion for interpreting ICC is that an ICC value of ≥0.75 is considered to be excellent/high reproducibility, ICC values in the range of 0.4 to 0.75 indicate fair to moderate reproducibility, and an ICC value of less than 0.4 indicates low to poor reproducibility [25]. In the present study, a threshold of an ICC value = 0.5 was used in order to confine our age- and GSR-related analyses to functional connections that were reasonably reliable. Within each group, multi-scan ICC values for specific correlations were variable, ranging from effectively zero to moderate/high. Some multi-scan ICC measures are negative, theoretically, due to relatively lower between-subject variability compared with within-subject variability. However, the reasons for negative ICC values are still unclear [26] and our analysis was based on all positive ICCs.

**Table 1:** Multi-scan ICC measures.

Correlations	Young	Old
**With GSR**		
All	0.32±0.14	0.27±0.16
Significant	0.35±0.15	0.32±0.16
Non-significant	0.30±0.14	0.26±0.16
Positive significant	0.38±0.14	0.34±0.16
Negative significant	0.28±0.13	0.24±0.14
**Without GSR**		
All	0.39±0.15	0.37±0.14
Significant	0.39±0.15	0.38±0.14
Non-significant	0.39±0.13	0.35±0.14
Positive significant	0.39±0.15	0.38±0.14
Negative significant	N/A	N/A

**Table 1** Listed are the mean and standard deviation of multi-scan ICCs given for all, significant, non-significant, positive significant or negative significant correlations for each group with and without GSR.

#### Age-related differences in reliability of RSFC

**Figure 1:**
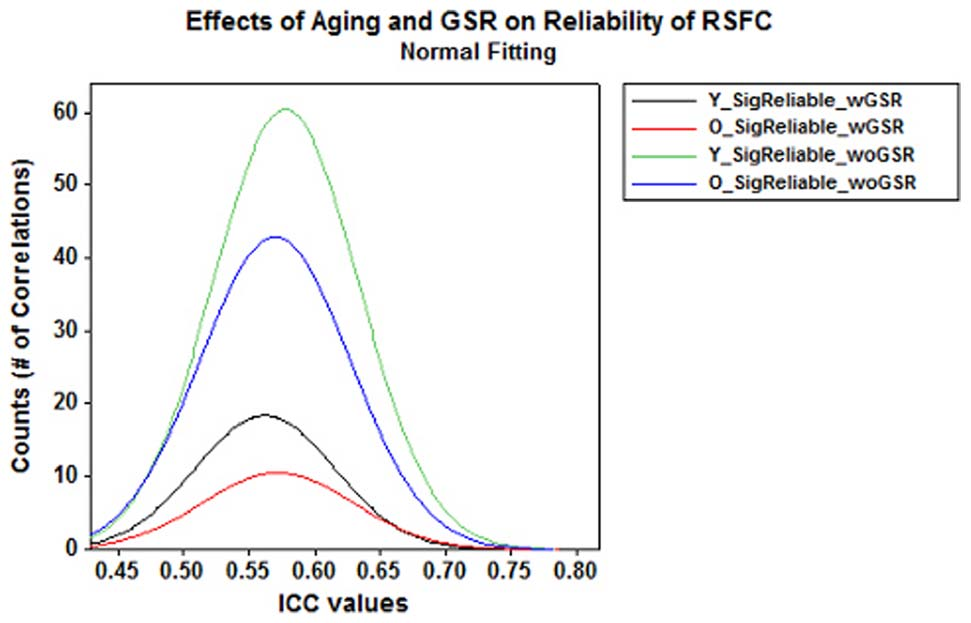
Effects of aging and GSR on reliability of RSFC. Frequency plots of multi-scan ICCs for significant and reliable correlations (i.e., *p*-value <0.05 adjusted by FDR correction, ICC >0.5) showed pronounced decreases in significant and reliable (SigRe) correlations with aging and GSR. (Y–Young, O–Old, wGSR–with GSR, woGSR–without GSR).

Using Binomial proportion test, significant (i.e., correlation is significant at the group level for each of the 3 scans, *p*-value <0.05 adjusted by FDR correction) and reliable (multi-scan ICC >0.5) correlations was compared between the two groups (**Figure 1** **;** **Table 2**). We found that significantly higher proportion of reliable correlations from the young group than from the old group with GSR (*p*-value = 0.028; **Figure S2**) and without GSR (*p*-value <0.001; **Figure S3**). The young group also showed significantly higher proportion of positive correlations as compared to the old group (*p*-value <0.001) both with and without GSR (**Table 3**).

**Table 2:** Effects of aging and GSR on significant and reliable correlations.

	Young	Old
**With GSR**		
All Significant	1477	1099
Significant and Reliable	246	153
Proportion of Sig. and Rel.	0.167	0.139
**Without GSR**		
All Significant	3478	3050
Significant and Reliable	842	618
Proportion of Sig. and Rel.	0.242	0.203

**Table 2** Binomial proportion tests demonstrated that 1) the young group had statistically more significant and reliable correlations (i.e., FDR corrected *p*-value <0.05, ICC value >0.5) than the old group with GSR (*p*-value = 0.028) and without GSR (*p*-value <0.001), and 2) higher proportion of significant and reliable correlations were found without GSR than with GSR in both groups (*p*-value <0.001).

**Table 3:** Effects of aging and GSR on positive significant correlations.

	Young	Old
Total number of correlations	4186	4186
**With GSR**		
Positive significant	1070	884
*Proportion of Positive Correlations*	*0.256*	*0.211*
Negative Significant	409	209
*Proportion of Negative Correlations*	*0.098*	*0.050*
**Without GSR**		
Positive significant	3648	3304
*Proportion of Positive Correlations*	*0.871*	*0.789*
Negative Significant	0	0

**Table 3** Binomial proportion test demonstrated that 1) both groups had higher proportion of positive significant correlations than negative significant correlations (*p*-value <0.001); 2) both groups had higher proportion of positive correlations without GSR than with GSR (*p*-value <0.001).

#### Significant versus non-significant correlations

Within each group, we tested the differences between significant and non-significant correlations using Wilcoxon rank-sum test, and we found that significant correlations were significantly more reliable than non-significant correlations (*p*<0.0001 for both groups with GSR (**Figure S4 a–b**). The Wilcoxon rank-sum test was also used to test multi-scan ICCs without GSR and it showed that significant correlations were significantly more reliable than non-significant correlations only for the old group (*p*<0.0001), and there was no significant difference between them in the young group (*p* = 0.36).

#### Positive versus negative correlations

**Figure 2:**
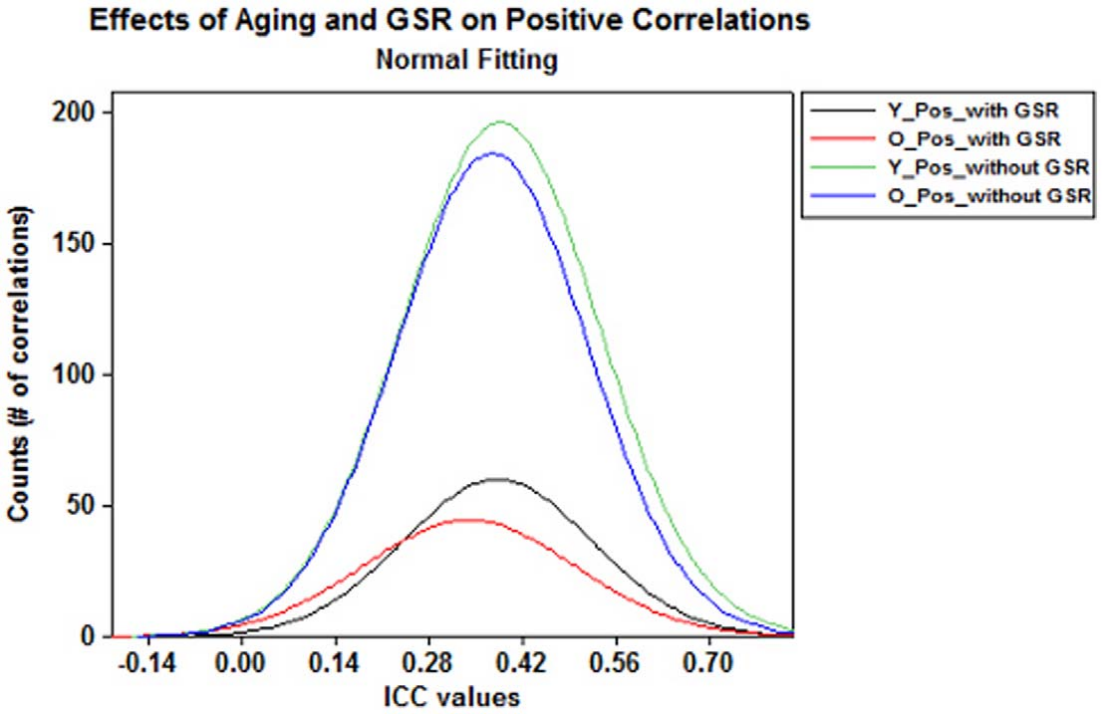
Effects of aging and GSR on positive correlations. Frequency plots of multi-scan ICCs for significant positive correlations showed pronounced decreases in positive (Pos.) correlations with aging and GSR.

Based on binomial proportion tests, we found significantly higher proportion of positive correlations than negative correlations in both groups with and without GSR (*p*<0.001 for all comparisons) (**Figure 2**). Negative correlations were found in both groups with GSR but none of them survived after FDR correction without GSR (**Figure S4 c–d**). The Wilcoxon rank-sum test suggested that multi-scan ICCs for positive correlations were significantly greater than for negative correlations (*p*<0.0001 for all comparisons) in both groups with GSR.

#### Reliability and magnitude of functional connections

**Figure 3:**
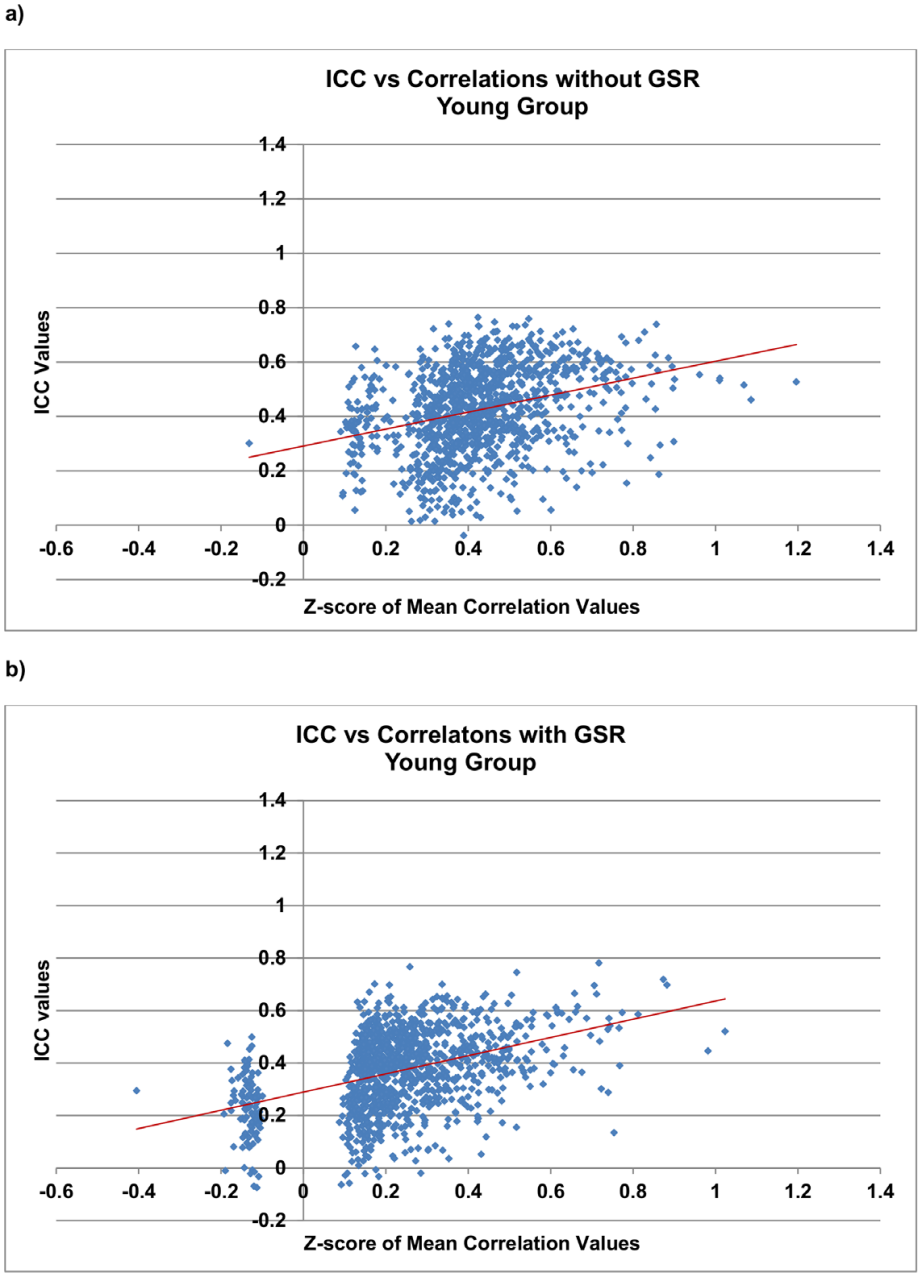
Reliability of RSFC *vs.* magnitude of functional connections in the young group. A left shift of data points indicates a reduction in the magnitude of functional connections when GSR is applied. The Wilcoxon signed-rank test shows reduced ICC values when GSR is applied (*p*-value <0.0001). Each data point represents a correlation that is significant both with and without GSR. Linear regression fits were overlaid on the data.

**Figure 4:**
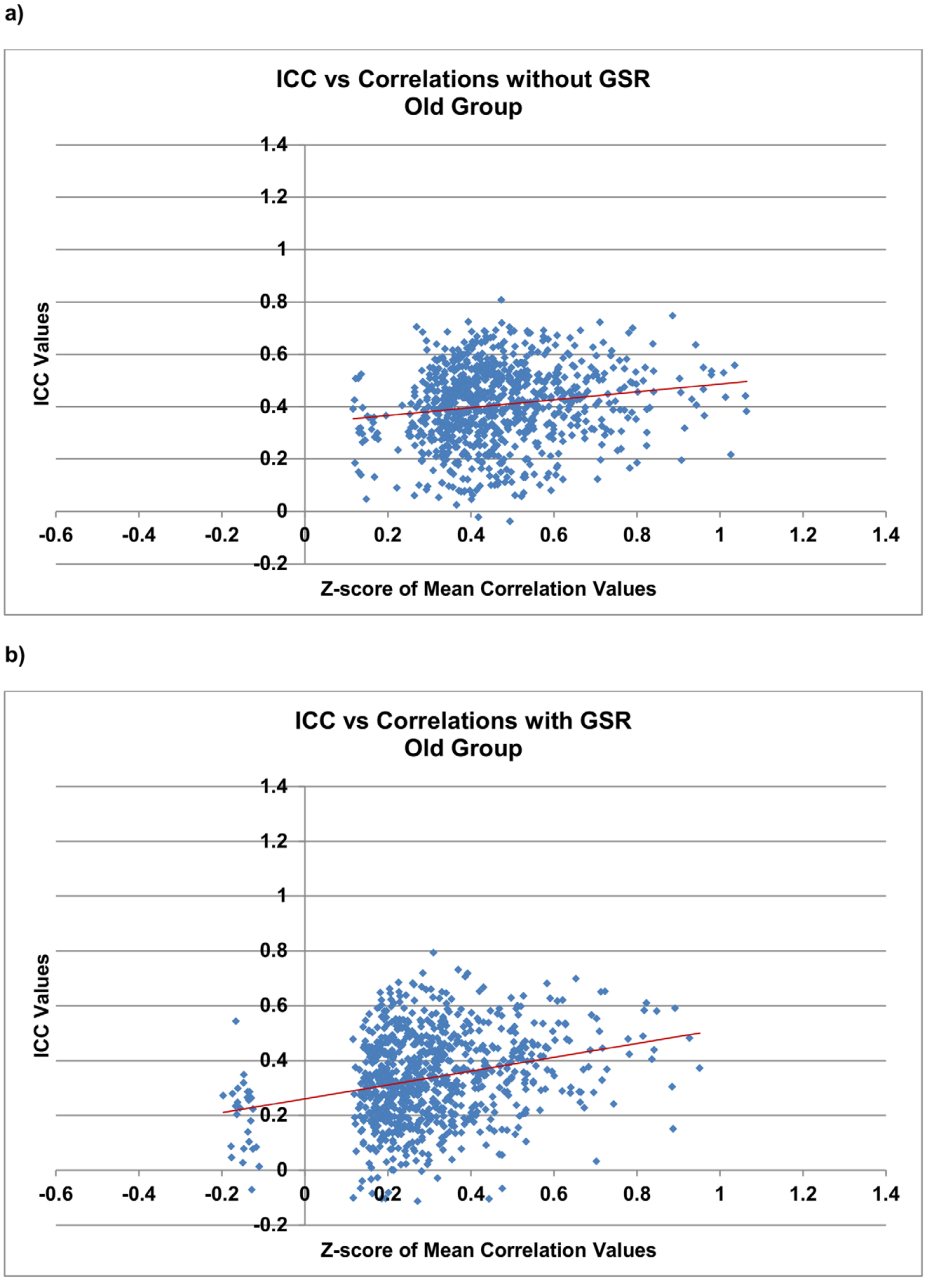
Reliability of RSFC *vs.* magnitude of functional connections in the old group. A left shift of data points indicates a reduction in the magnitude of functional connections when GSR is applied. The Wilcoxon signed-rank test shows reduced ICC values when GSR is applied (*p*-value <0.0001). Each data point represents a correlation that is significant both with and without GSR. Linear regression fits were overlaid on the data.

For each functional connection that was significant both with and without GSR, its magnitude (i.e., group-averaged multi-scan correlation coefficients) was Fisher *z*-transformed and plotted against the corresponding ICC value shown in **Figure 3** **and** **4** for each group. Linear fitting revealed a trend of higher correlations leading to higher ICC measures (**Figure 3** **–** **4** **, S5**). Within each group, we carried out a Wilcoxon signed-rank test on the ICC values of these matched pairs of significant correlations. The results showed that ICC values were significantly greater without GSR (*p*-value <0.0001). Within each group, a “left shift” of correlations was observed when GSR is applied (**Figure 3b** **and** **Figure 4b**), indicating reduced magnitude of correlations due to GSR.

#### Global signal regression factor in reliability of RSFC

**Figure 5:**
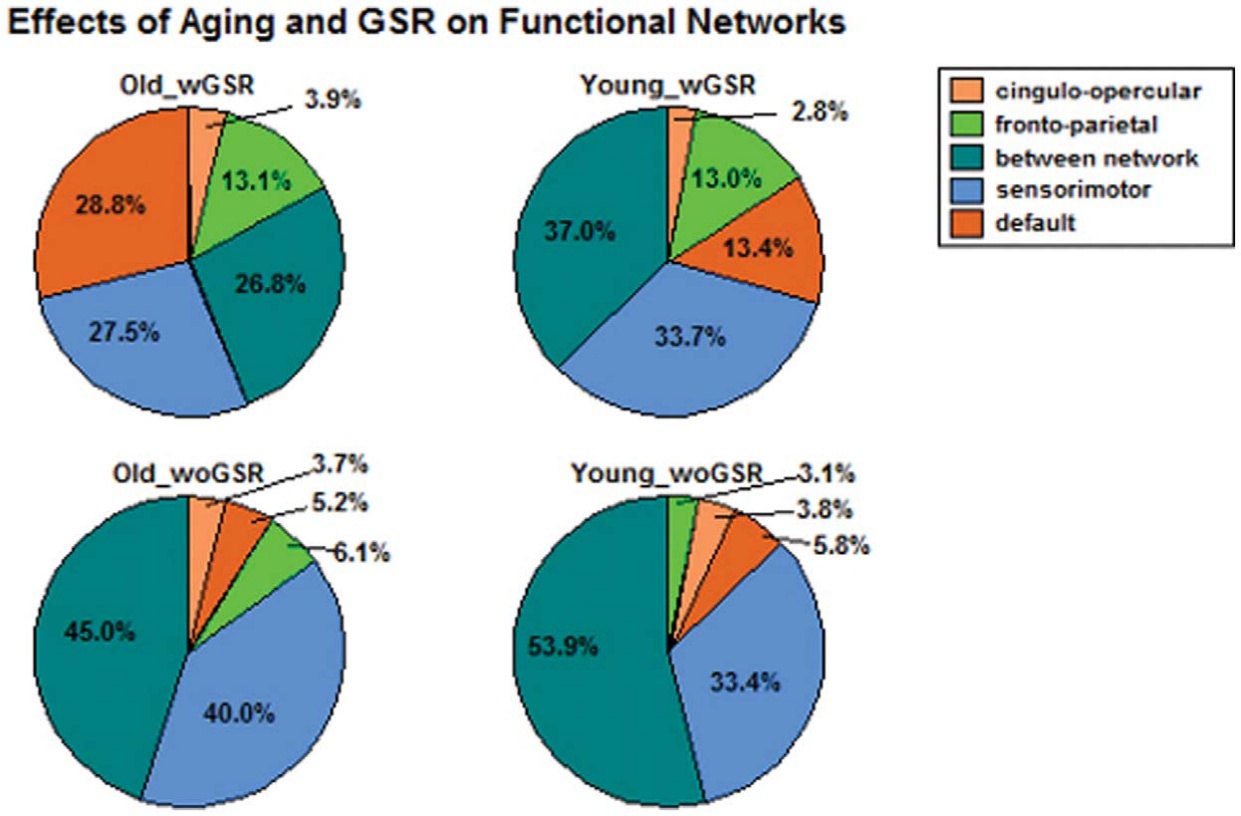
Effects of aging and GSR on brain functional networks. Pie chart illustrates the proportion of significant and reliable functional connections within each network. Binomial proportion tests showed aging was associated with significant decreases in reliable connections between-network but increases within sensorimotor and fronto-parietal networks. Each percentage number indicates the proportion of functional connections within corresponding networks.

**Figure 6:**
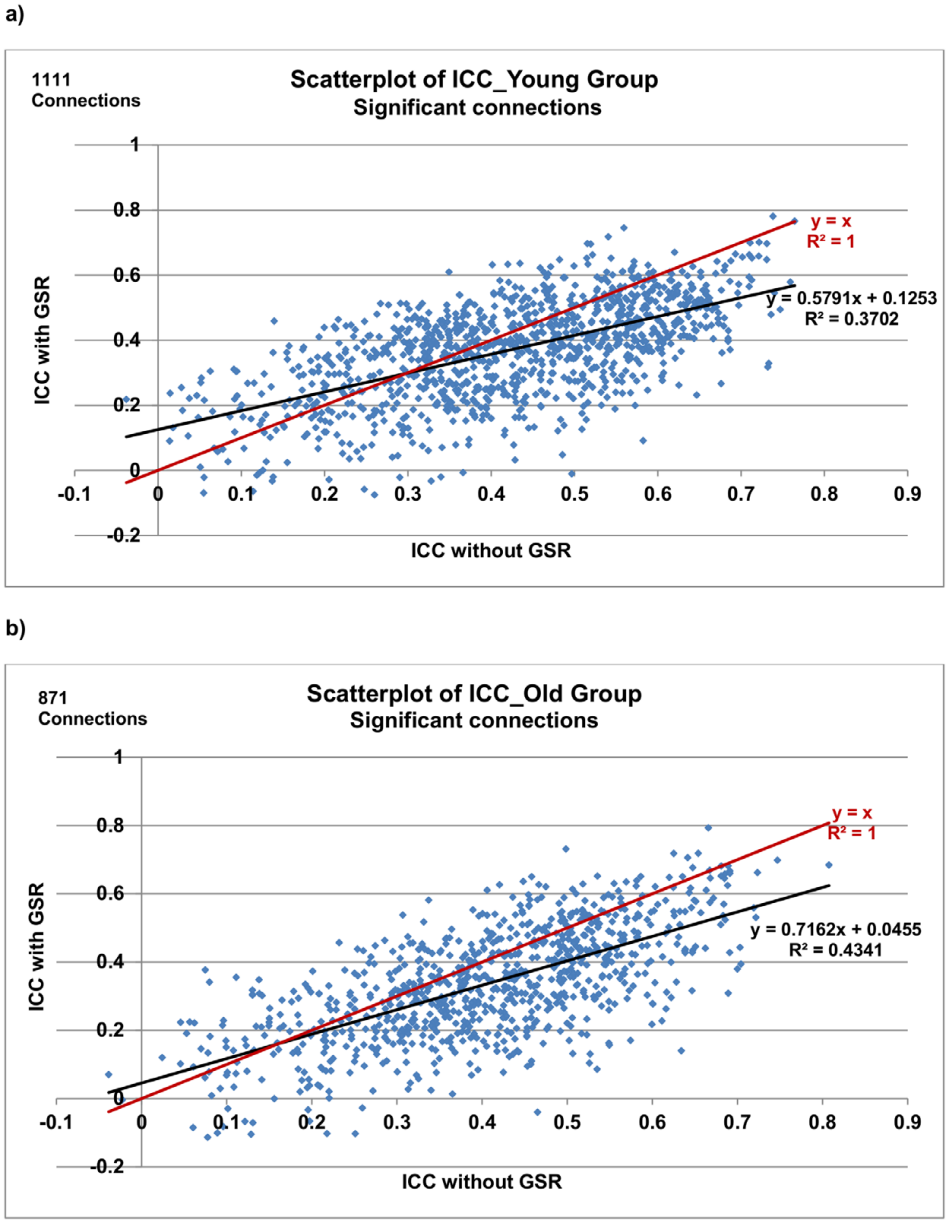
Scatterplot of ICC for connections with vs. without GSR. GSR tends to reduce the reliability of connections (i.e., regression lines are under y = x when ICC >0.3 for the young group and ICC >0.15 for the old group). Each data point indicates a significant connection from each group. Black lines represent linear regression fits of the data and red lines represent y = x.

Across all the connections between ROIs, using Binomial proportion test, we found significantly more reliable correlations without GSR than with GSR in both groups (*p*-value <0.001, **Figure 1**, **S2–S3;** **Table 2**), and significantly more positive correlations without GSR than with GSR (*p*-value <0.001, **Figure 2** **;** **Table 3**). Within default and fronto-parietal network, we found significantly more reliable correlations with GSR than without GSR in both groups. However, it also showed significantly less reliable correlations between-network with GSR than without GSR in both groups (*p*-value <0.001, **Figure 5** **;** **Table 4**). A direct comparison of ICC values with *vs.* without GSR was shown in **Figure 6**. ICC values for those reliable correlations (ICCs >0.5) were reduced after GSR (i.e., regression lines were underneath the y = x line for ICCs >0.5.).

**Table 4:** Effects of GSR on brain functional networks.

Network	Young		Old	
	Binomial Proportion Test	*p*-value (CI = 95%)	Binomial Proportion Test	*p*-value (CI = 95%)
Default	wGSR>woGSR	0.001	wGSR>woGSR	<0.001
Fronto-parietal	wGSR>woGSR	<0.001	wGSR>woGSR	0.008
Sensorimotor	Not equal	0.915	wGSR<woGSR	0.001
Cingulo-opercular	Not equal	0.444	Not equal	0.909
Between-network	wGSR<woGSR	<0.001	wGSR<woGSR	<0.001

**Table 4** Binomial proportion test showed the proportion of reliable functional connections was significantly affected by GSR both within- and between-network.

#### Network analysis of significant and reliable correlations

Among 92 ROIs, 17 in the fronto-parietal network, 19 are in the default-mode network, 23 in the cingulo-opercular network and 33 in the sensorimotor network (**Figure S1**; **Table S1**). We found 1) highest number of significant and reliable correlations between regions in the sensorimotor network (**Figure 5** **;** **Table 5**), 2) least number of significant and reliable correlations between regions in the cingulo-opercular network in both groups with and without GSR (**Figure 5** **;** **Table 5**), 3) the old group had significantly less number of reliable correlations between-network than the young group with and without GSR (*p*-value <0.001, **Figure 5** **;** **Table 6**), 4) the old group had significantly more reliable correlations within fronto-parietal and sensorimotor networks without GSR than the young group (*p*-value <0.006, **Figure 5** **;** **Table 6**).

**Table 5:** Network analysis of significant and reliable correlations.

Network	Young	Old
**With GSR**		
Total # of Sig. Re. Corr.	246	153
Default	33 (13.4%)	44 (28.8%)
Fronto-parietal	32 (13.0%)	20 (13.1%)
Sensorimotor	83 (33.7%)	42 (27.5%)
Cingulo-opercular	7 (2.8%)	6 (3.9%)
Between-network	91 (37.0%)	41 (26.8%)
**Without GSR**		
Total # of Sig. Re. Corr.	842	618
Default	49 (5.8%)	32 (5.2%)
Fronto-parietal	26 (3.1%)	38 (6.1%)
Sensorimotor	281 (33.4%)	247 (40.0%)
Cingulo-opercular	32 (3.8%)	23 (3.7%)
Between-network	454 (53.9%)	278 (45.0%)

**Table 5** Binomial proportion test demonstrated that 1) the young group had significantly more between-network correlations than the old group with and without GSR (*p*-value <0.015), and 2) both groups had significantly higher proportion of between-network correlations without GSR than with GSR (*p*-value <0.001). Listed in parentheses are proportions of significant and reliable correlations observed in each network.

**Table 6:** Effects of aging on brain functional networks.

Network	Binomial Proportion Test	*p*-value (CI = 95%)
**With GSR**		
Default	Old>Young	<0.001
Fronto-parietal	Not equal	0.985
Sensorimotor	Not equal	0.181
Cingulo-opercular	Not equal	0.570
Between-network	Old<Young	0.015
**Without GSR**		
Default	Not equal	0.594
Fronto-parietal	Old>Young	0.004
Sensorimotor	Old>Young	0.005
Cingulo-opercular	Not equal	0.938
Between-network	Old<Young	<0.001

**Table 6** Binomial proportion test showed the old group had significantly more reliable correlations within network than the young group and less reliable correlations between-network.

#### Group-level consistency across scans

**Figure 7:**
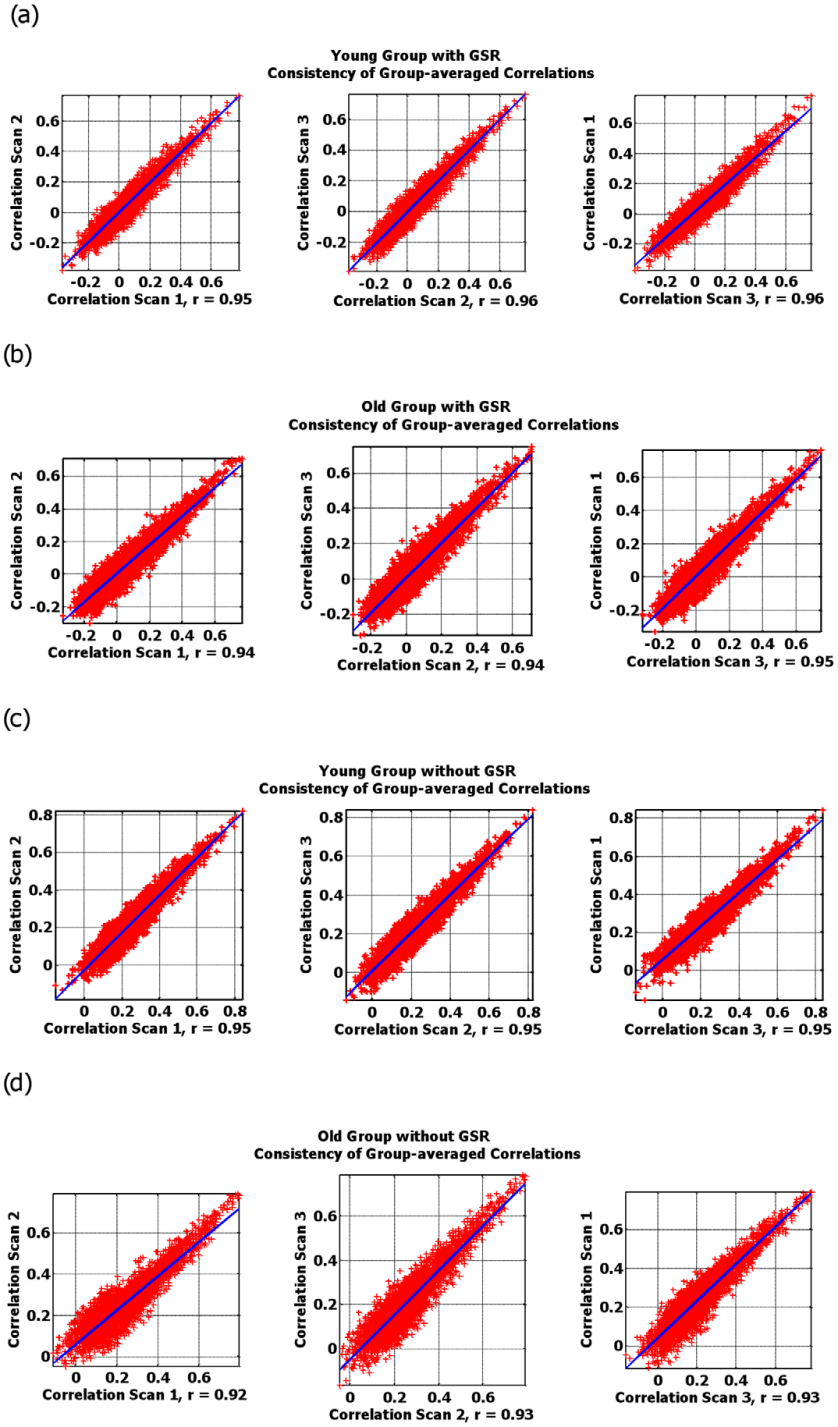
Scan 1 *vs.* scan 2 *vs.* scan 3. Group-averaged correlations from each scan session are plotted against each other. High consistency of RSFC from scan to scan is observed independent of aging and GSR factor. Overlaid blue lines represent linear regression fits of the data points and the *r*-values of the fit represent Pearson correlations of the data points.

We also assessed the consistency of group-averaged functional connectivity across scans. Group-level correlation matrices were obtained by averaging all *z*-transformed correlation coefficients across all subjects for each scan and each group. The reverse transformation was run on each resulting mean correlation coefficient, resulting in the creation of three 1 by 4186 matrices of correlation coefficients for each group. Group-averaged correlations for all three scans were then plotted against each other and the results exhibited high consistency between any two of them for both groups (**Figure 7**).

### Stability of Functional Connectivity

To investigate the stability of RSFC, we measured the consistency of correlations within and between subjects using Kendall’s *W*. More specifically, within each subject the rank order of correlations was evaluated across scans and within each scan session the rank order of correlations was evaluated across subjects. Within-subject across scans, Kendall’s *W* measures were variable, ranging from moderate to high (**Table 7**). Between subjects within each scan, the measures were variable and much lower than the measures within-subject Kendall’s *W* (**Table 8**).

**Table 7:** Kendall’s *W* within-subject across scans.

Functional Connections	Young	Old
**With GSR**		
All	0.67±0.06	0.64±0.06
Significant	0.77±0.05	0.76±0.04
Non-significant	0.56±0.07	0.54±0.06
Positive significant	0.66±0.04	0.66±0.04
Negative significant	0.52±0.08	0.48±0.07
**Without GSR**		
All	0.67±0.06	0.63±0.06
Significant	0.65±0.06	0.63±0.06
Non-significant	0.56±0.08	0.50±0.07
Positive significant	0.65±0.06	0.63±0.06
Negative significant	N/A	N/A

**Table 7** Listed are the mean and standard deviation of Kendall’s *W* within subject across scans for all, significant, non-significant, positive significant and negative significant correlations for both groups.

**Table 8:** Kendall’s *W* between-subject within scan.

Functional Connections	Young				Old			
With GSR	Scan 1	Scan 2	Scan 3	Mean	Scan 1	Scan 2	Scan 3	Mean
All	0.30	0.30	0.32	0.31	0.32	0.29	0.30	0.30
Significant	0.50	0.49	0.52	0.50	0.51	0.48	0.49	0.49
Non-significant	0.10	0.10	0.12	0.11	0.13	0.12	0.11	0.12
Positive significant	0.23	0.23	0.23	0.23	0.26	0.24	0.24	0.25
Negative significant	0.06	0.06	0.06	0.06	0.05	0.03	0.03	0.04
**Without GSR**								
All	0.27	0.28	0.28	0.28	0.29	0.25	0.27	0.27
Significant	0.24	0.24	0.25	0.24	0.25	0.24	0.24	0.24
Non-significant	0.08	0.06	0.06	0.06	0.07	0.07	0.05	0.06
Positive significant	0.24	0.24	0.25	0.25	0.25	0.24	0.24	0.24
Negative significant	N/A	N/A	N/A	N/A	N/A	N/A	N/A	N/A

**Table 8** Listed are the mean and standard deviation of Kendall’s *W* between subjects within scan for all, significant, non-significant, positive significant and negative significant correlations.

#### Significant versus non-significant RSFC within subjects across scans

We compared the Kendall’s *W* for significant and non-significant correlations within each subject across all three scans (**Figure S6**). *The Wilcoxon rank-sum* test demonstrated that significant correlations were significantly more stable than non-significant correlations for both groups with and without GSR (*p*-value <0.0001 for all comparisons).

#### Positive versus negative RSFC within subjects across scans

The Wilcoxon rank-sum test demonstrated that positive correlations were significantly more stable than negative correlations for both groups with GSR (*p*-value <0.0001 for all comparisons). No negative correlations were observed without GSR.

#### Age-related differences in stability of RSFC

**Figure 8:**
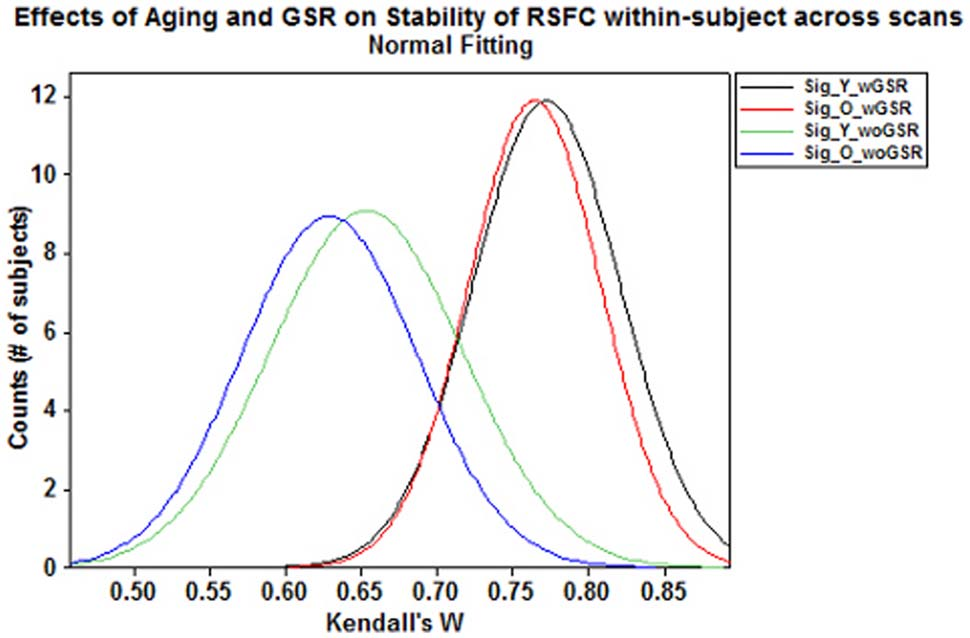
Effects of aging and GSR on stability of RSFC within-subject across scans. Frequency plots of Kendall’s *W* across scan sessions for significant correlations showed pronounced decreases in Sig. correlations with GSR. High stability of RSFC from scan to scan remains independent of aging effects.

Using Wilcoxon rank-sum test, we found no significant difference in stability of significant correlations between the two groups (*p*-value = 0.572 with GSR and *p*-value = 0.136 without GSR, **Figure 8**). Similarly, no significant differences in positive correlations were found between groups (*p*-value = 0.794 with GSR and *p*-value = 0.115 without GSR).

#### Global signal regression factor in stability of RSFC

The Wilcoxon rank-sum test suggested that Kendall’s *W* within-subject across-scan for significant correlations with GSR was significantly greater than without GSR for both groups (*p*-value <0.0001; **Figure 8**). For positive correlations, the old group showed significantly higher stability with GSR than without GSR (*p*-value = 0.024) but no significant differences in the young group (*p*-value = 0.768).

#### Stability between subjects within scan

**Figure 9:**
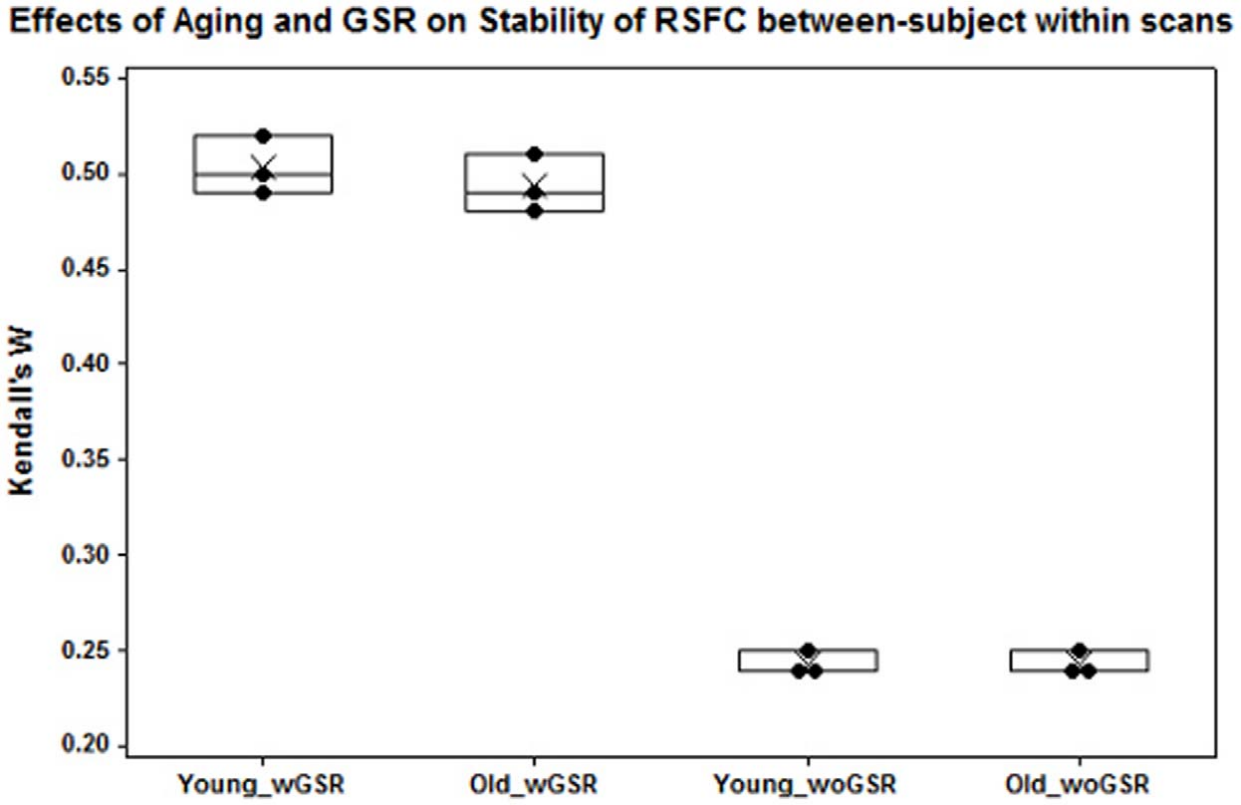
Effects of aging and GSR on stability of RSFC between-subject within scan. GSR could potentially enhance the agreement/stability across scan sessions from subject to subject. Each black dot represents Kendall’s *W* between subjects within each scan. Cross points represent Kendall’s *W* values averaged across three scans.

We found the stability of RSFC for each scan was highly similar between subjects within scan (**Figure 9**) but much lower as compared to the stability of RSFC within subject across scans (**Table 7** **–** **8**). Higher stability of RSFC between subjects within scan was observed with GSR than without GSR (**Figure 9** **, Figure S7;** **Table 8**).

## Discussion

Growing evidence suggests that human brains undergo dynamic functional reorganizations during the lifespan. Resting-state functional connectivity provides insight into this large scale structural interaction between brain regions. The goal of the current study was to investigate age-related differences in reliability and stability of resting-state functional connectivity across scan sessions. We found that RSFC is more reliable for the young group and highly consistent for both groups, and that, consistent with previous studies [12], [24], [27], [28], regressing the global signal altered group differences of between-region functional connections.

### Reliability

#### Highest reliability of RSFC in significant correlations

Multi-scan ICCs for statistically significant and positive significant correlations with GSR across subjects in each group exhibited higher degree of test-retest reliability compared with non-significant, and/or negative significant correlations. **Table S2** and **S3** display the significant and reliable functional connectivity measures which were observed to be part of the same functional networks, or corresponding with task-evoked activations. For example, we observed the most highly reliable (multi-scan ICC = 0.81) correlations between regions of left parietal and right precentral gyrus which were part of sensorimotor network in the old group without GSR. Correspondingly, correlations between regions of dorsal frontal cortex (dFC) and regions of inferior parietal lobe (IPL) as part of the fronto-parietal network were highly reliable (multi-scan ICC = 0.76) in the young group without GSR. Regions of the right thalamus and left thalamus which were part of cingulo-opercular network were highly correlated (multi-scan ICC = 0.78) in the young group with GSR. Regions of right angular gyrus and left posterior cingulate cortex, part of default-mode network, exhibited highly reliable correlations (multi-scan ICC = 0.79) in the old group with GSR. These results are in agreement with task-evoked activations which have been demonstrated in previous studies [1], [15], [29]–[32].

#### Age-related differences in reliability of RSFC

Using the Binomial proportion test, we found significantly higher proportion of reliable correlations in the young group than in the old group across 92 ROIs. To further examine this difference in each correlation, we compared the RSFC within- and between-network and found that pronounced decreases in the proportion of reliable correlations between-network in the old group with and without GSR (*p*-value <0.001, **Table 5** **–** **6**). However, the old group also showed pronounced increases in the proportion of reliable correlations within-network especially without GSR (*p*-value <0.006, **Table 5** **–** **6**).

These findings suggest between-network connections may be more vulnerable to aging effects than within-network connections. This is consistent with our recent work using support vector machines which showed that between-network connections best differentiated older adults from younger adults based on their resting state functional connectivity [33]. Other studies have shown aging is associated with decreased functional connectivity in the DMN involving the superior and middle frontal gyrus, posterior cingulate cortices, and the superior parietal region [2], [20], [34]–[35]. Interestingly, we observed a significant increase in reliable connections in the DMN in the old group with GSR, whereas no significant differences were found in terms of the proportion of reliable correlations between the young and the old group without GSR. This further confirmed that group differences could be altered, or perhaps misinterpreted, after GSR [28].

#### GSR-related differences in reliability of RSFC

Binomial proportion test showed that the proportion of significant and reliable connections is significantly decreased with GSR in both groups (**Figure 1**, **S2–S3**; **Table 2**). Similarly, we observed significantly more positive correlations without GSR than with GSR in both group (**Figure 2**, **Table 3**). We also found that negative correlations were only present with GSR, exhibiting relatively low reliability (mean multi-scan ICCs <0.3) and stability (multi-scan Kendall’s *W* within subjects <0.6 and between subjects within scan ≤0.06).These findings not only suggest higher reliability of RSFC without GSR than with GSR but also further confirms the observations in previous studies that GSR reduces the sensitivity for detecting true correlations [27] as well as negatively biases the correlations [24] and could fundamentally alter inter-regional correlations [28], especially when examining group differences.

### Stability

#### Age-related differences in stability of RSFC

We examined all connections between ROIs across the pre-defined 92 seed regions using Wilcoxon rank-sum test. A high degree of multi-scan stability of RSFC within subjects was found in both groups and no significant difference between groups was observed (*p*-value = 0.572 with GSR and *p*-value = 0.136 without GSR). This suggests that functional connectivity observed from each subject is highly stable or consistent from scan to scan and this stability/consistency remains independent of aging effects. We also observed relatively lower Kendall’s *W* scores across all correlations within each scan between subjects (**Table 8**), suggesting high individual variability exists in RSFC.

#### GSR-related differences in stability of RSFC

Although GSR significantly reduced the number of reliable connections in both groups (**Figure S2–S3**; **Table 2** **–** **3**) especially those between-network connections (**Table 4**), it elevated the observed stability of RSFC both within-subject across scans (**Figure 8** **;** **Table 7**) and between subjects within scan (**Figure 9** **;** **Table 8**). These findings suggest GSR could potentially alter the pattern of RSFC in each observation from each subject and each scan and enhance the agreement/stability within subject from scan to scan or within scan from subject to subject. Furthermore, our results showed highly stable RSFC within each individual across scan sessions (**Age-related differences in stability of RSFC**), suggesting highly consistent measurements of RSFC independent of aging factor. However, measures between subjects within each scan were relatively low, indicating the existence of individual variation. It may imply that variations of RSFC between individuals need further examination in order to discriminate from neuropathological changes.

### Functional Brain Network with Normal Aging

Normal aging is associated with anatomical and functional changes as well as cognitive decline [34], [36]. However, the effect of aging on brain functional connectivity remains largely unknown. In the present study, we found fewer significant connections between ROIs from 92 pre-defined seed regions in the old group than in the young group. This is consistent with the observation made by Wu et al. [19], who investigated changes in small-world and modular organization of structural brain network with normal aging, demonstrating a notable decrease in the connector ratio and the intermodule connections in the old group.

Conversely, we observed concomitant age-related increases in reliable functional connections within sensorimotor and fronto-parietal networks. The old group showed significantly higher proportion of reliable connections than the young group without GSR within these two networks (**Figure 5** **;** **Table 5** **–** **6**). This could be potentially explained by a compensation mechanism that the decline of reliable functional connections between regions with aging, especially the long range connections between networks, is compensated by increased functional connections within networks.

In a recent study, Tomasi et al. [37] evaluated age-related effects on functional brain networks based on a sample of 913 healthy subjects using functional connectivity density mapping. In their study, global signal intensity was normalized across time points. They found that aging was associated with increases in long-range functional connectivity density in somatosensory and subcortical networks and pronounced decreases in the DMN and dorsal attention network. In our study, we also found that aging was associated with significant increases in proportion of functional connections in the sensorimotor network, whereas no significant differences were found in DMN between the two age groups without GSR (**Figure 5** **;** **Table 5** **–** **6**). Interestingly, with GSR, there was increased proportion of reliable connections in DMN but no significant difference in the somatosensory network in the old group. This further draws attention to the issue of how global signal should be handled in data pre-processing in order to avoid the potential misinterpretation of group differences at functional network level. Research has shown an age-related reduction in occipital activity coupled with increased frontal activity, which is known as posterior-anterior shift in aging (PASA) [38], and age-related increases and correlations with parietal activity. In our study, occipital was not covered by the pre-defined seed regions, however, we observed increases in reliable connections in fronto-parietal network with aging without GSR (**Figure 5** **;** **Table 5** **–** **6**). The frontal-parietal network is engaged by a wide range of higher level cognitive tasks and is thought to be involved in active and adaptive task control [39]. This increase in reliable connections within frontal-parietal network might compensate the age-related decline in adaptive task control.

The cingulo-opercular network is engaged in a variety of tasks and thought to contribute to the flexible control of human goal-directed behavior and affect downstream sensorimotor processing through the stable task-set maintenance [39]. We observed the least number of reliable connections within cingulo-opercular network in both groups without age-related differences (**Table 5** **–** **6**), suggesting dynamic changes in functional connections within this network throughout adults’ lifespan.

Additionally, GSR plays a significant role in the observed RSFC. We conclude that GSR reduces the overall reliability of RSFC (**Figure 1** **–** **2** **;** **Table 1** **,** **2** **,** **3**) but increases the stability in both age groups (**Figure 8** **–** **9** **;** **Table 7** **–** **8**), and that GSR affects the interpretation of group differences, especially, in each brain network presented in this study (**Figure 5** **;** **Table 4**).

Several alternatives to global signal regression have been proposed such as applying principal components analysis (PCA) to resting-state fMRI time-series in order to regress out the component most correlated with signal of interest [40]–[41]. Our future work will determine the test-retest reliability of connectivity measures using PCA.

### Limitations

The age-related differences between young and old groups, presented in this study, were based on resting-state functional connectivity among 92 pre-defined seed regions. The axial slice acquisition of the fMRI data prevented the inclusion of the occipital and cerebellar networks in our analysis, as several subjects included in the ICBM dataset did not have coverage in these regions. The use of ROIs across the whole brain would have been ideal in that we would be able to assess the reliability of each correlation drawn from an even larger sample, however, the statistical tests presented in our study still showed significant age-related differences in RSFC.

Group differences in head movement have been shown by Van Dijk et al. [42] to significantly affect correlations between seed regions. Given our recent work [33], using the same ICBM dataset, showing that significantly more motion in the old group, it should be noted that the motion in older subjects was fairly constant from time to time point with no large spikes of motion present. Interestingly, after removal of motion-sensitive correlations, more than 70% of total correlations were preserved, suggesting that motion may account for some of the differences in connectivity observed in this study but the overall results should be robust based on Binomial proportion test and Wilcoxon rank-sum test.

In the present study, we examined how GSR affects RSFC primarily due to the ongoing debate on the use of GSR when studying RSFC. We found GSR reduces the overall reliability in young and old groups, reduces the magnitude of correlations and could potentially alter group differences. As shown in **Figure 3** **,** **4** **and S5,** a higher correlation leads to a higher reliability score. Therefore, the reduced reliability score (i.e., ICC value) for a functional connection, when global signal is removed, might be confounded by the reduced correlations due to GSR. But overall, GSR, as a factor of reducing correlation values, reduces ICC measures and thus the reliability of functional connections. Other factors such as the magnitude of noise and/or motion which affect correlation values could also potentially have an impact on ICC measures.

## Materials and Methods

### Participants

Resting-state fMRI data were obtained from 29 younger adults (18–35 years, mean age = 25.8 years; 13 males/16 females) and 26 older adults (55–85 years, mean age = 64.7 years; 11 males/15 females). All participants are right-handed.

### fMRI Data Acquisition

Three resting-state scans from each subject were acquired using a 3.0 Tesla scanner. Each scan consisted of 128 gradient-echo EPI functional volumes (TR = 2.0 seconds; 64×64 matrix, 23 axial slices). For 19 out of the 29 younger adults, two scans were acquired with voxel size 4×4×5.5 mm^3^ and the third one was 4×4×4 mm^3^, while the other 10 subjects had all three scans with voxel size 4×4×4 mm^3^. 20 out of 26 older adults, two scans were acquired with voxel size 4×4×5.5 mm^3^ and the third one was 4×4×4 mm^3^, while the other 6 subjects had all three scans with voxel size 4×4×4 mm^3^. All individuals were asked to keep their eyes closed during the scan. As stated earlier, resting-state fMRI data presented in our study were obtained from the International Consortium for Brain Mapping (ICBM) dataset which were made publically available in the 1000 Functional Connectomes project www.nitrc.org/projects/fcon_1000. Each contributor’s respective ethics committee approved submission of deidentified data. The institutional review boards of NYU Langone Medical Center and New Jersey Medical School approved the receipt and dissemination of the data.

### fMRI Data Preprocessing

Data were preprocessed using scripts slightly adapted from fcon_1000 using a combination of AFNI (version AFNI_2009_12_31_1431, http://afni.nimh.nih.gov/afni) and FSL (version 4.1.4, www.fmrib.ox.ac.uk/fsl). Data were first deobliqued and reoriented to RPI orientation for use in FSL. AFNI was then used to perform the initial preprocessing steps of 1) slice time correction for interleaved acquisition using Fourier-space time series phase-shifting, 2) motion correction to the average of the time series by aligning each volume to the mean image using Fourier interpolation, 3) skull stripping, and 4) selecting the eighth image for subsequent use in co-registration of BOLD images to the high resolution anatomic image. Further data preprocessing was carried out using FSL and comprised 5) spatial smoothing using a Gaussian kernel with FWHM = 6 mm, and 6) normalizing all volumes by a single scaling factor. Band-pass filtering (0.005–0.1 Hz) and detrending to remove linear and quadratic trends for each subject were then performed using AFNI. Masks of preprocessed data for each subject were generated using FSL. Functional data were then transformed into MNI152 (Montreal Neurological Institute 152-brain template; voxel size = 3×3×3 mm^3^) space using a three-step process: First, a 6 degree of freedom linear affine transformation was carried out using FLIRT [43]–[44] to align the functional data into structural space. The anatomical image was then aligned to the standard MNI152 space using a 12 degree of freedom linear affine transformation using FLIRT. The resulting transformation was then applied to each subject’s functional dataset.

### Nuisance Signal Regression

Signal associated with nuisance covariates consisting of global signal, white matter (WM), CSF and 6 motion parameters obtained during the motion correction step were removed from the resulting preprocessed fMRI time-course data. WM and CSF masks were created by segmentation of each subject’s structural images and then applied to functional images to extract the WM and CSF signals. The global signal was calculated by averaging across all voxels in the brain. Following the regression of nuisance covariates, the residual signals were demeaned and re-sampled to the standard MNI-152 space (voxel size = 3×3×3 mm^3^).

One goal of the current study was to examine how global signal regression (GSR) affected the reliability and stability of RSFC; therefore we pre-processed the data in two parallel analyses, with and without global signal regression (GSR).

### Resting-state Functional Connectivity

#### Regions of interest

92 spherical ROIs with radius 5 mm (**Figure S1**; **Table S1**), derived from several meta-analyses of task-related fMRI studies previously defined by Dosenbach et al. [45], were selected in our study. Dosenbach et al. extracted time series from 160 seed regions covering several networks. Due to axial slice selection, not all subjects from ICBM datasets had EPI coverage of the occipital lobe and cerebellum, which resulted in 92 seed regions mainly consisting of default-mode, cingulo-opercular, fronto-parietal and sensorimotor networks. Some subjects had no signal in these areas and therefore were not included in our study.

### Statistical Methodology

#### Functional connectivity

Mean time series from each ROI were extracted, imported into MATLAB (R2010a, Mathworks), and correlated with that from every other ROI. Pearson correlation coefficients were calculated for each pair of regions for each subject and each scan. The resulting correlation coefficients were then *z*-transformed for calculations of multi-scan intraclass correlation coefficient (ICCs), or transformed into a distance measure in order to calculate Kendall’s coefficient concordance (Kendall’s *W*).

To determine the significance of each correlation, a one-sample *t*-test was run on the *z*-transformed correlation coefficients for both young and old group. Group-level significance of each correlation was defined by a two-sided *p*-value of 0.05, which was then adjusted for multiple comparisons using false discovery rate (FDR) correction (total 4186 correlations). More specifically, a significant correlation needs to be significant at the group level for each of the 3 scans with *p*-value <0.05 adjusted by FDR correction. Within each group, positive correlations were determined by a right-tailed one-sample *t*-test with a *p*-value of 0.05 adjusted by FDR correction. Similarly, negative correlations were determined by a left-tailed one-sample *t*-test with a *p*-value of 0.05 adjusted by FDR correction.

#### Reliability of functional connectivity

We attempted to examine if significant connections observed in a first scan session would be reproducible within-subject in the following sessions and any changes between-subject would be due to subject difference. Intraclass correlation is defined as a ratio of the variance of interest over the sum of the variance of interest plus error [46], and has been frequently used to measure test-retest reliability in fMRI data [12]–[13], [47]–[48]. We calculated the third ICC values defined by Shrout and Fleiss [46] as follows:
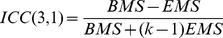
(1)where *BMS* is between-subjects mean square, *EMS* is residual/error mean square, and *k* is the number of scans per subject, which is 3 in our case.

Theoretically, high ICC values suggest that compared to between-subject variability (i.e., BMS), within subject variability (i.e., EMS) across scans is relatively smaller, indicating high consistency or reliability of within-subject RSFC.

Given multi-scan ICC for each correlation, we examined the effect of the following factors on the multi-scan reliability of RSFC. 1) Statistically significance: significant correlations were compared with non-significant correlations. 2) Patterns: significantly positive correlations were compared with that of significantly negative correlations. 3) Age: significant and reliable correlations in the young group were compared with those in the old group; 4) GSR: significant correlations obtained with GSR were compared with those without GSR within each group.

#### Stability of functional connectivity

We used Kendall’s coefficient concordance (Kendall’s *W*) to estimate if overall connections were consistent across subjects and across scans. Kendall’s *W* is a measure of agreement among raters based on ranks rather than values, and has been used to assess the concordance of time courses within an individual using fMRI data [49]–[50]. We calculated Kendall’s *W* as follows:
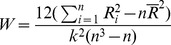
(2)where *k* is the number ofscans or number of subjects, *n* is the number of correlations, *R_i_* is the sum rank of the *i*th correlation across scans or subjects, 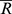
is the mean of *R_i_*’s. Kendall’s *W* reflects the agreement in the rank order of correlations across subjects or across scans. In other words, it indicates how stable RSFC is within-subject across scans or within-scan across subjects. As with ICCs, we examined the effect of the following factors on the multi-scan stability of RSFC: statistically significance, patterns, age, and GSR.

#### Statistical tests

With Binomial proportion test, one can test the hypothesis of the equality of two binomial proportions. In our study, it was used to test the proportion of significant correlations versus non-significant, positive versus negative correlations between the young and the old group. Significant and non-significant correlations are treated as binomial measures (i.e., 1′s and 0′s respectively) and we tested the proportion of 1′s in one group versus that in the other group (i.e., young vs. old).

Within each group, we tested the differences between significant and non-significant as well as between positive and negative correlations using Wilcoxon rank-sum test, which is a nonparametric test allowing to test measures of ICC or Kendall’s *W* that are not normally distributed.

## Supporting Information

Figure S1 Shown are 92 regions of interest (ROIs) used in this study taken from Dosenbach et al. (2010). All 92 ROIs are displayed on a surface rendering of the brain (ICBM 152) visualized with the BrainNet Viewer (http://www.nitrc.org/projects/bnv/). Red dots represent the ROIs from the default mode network, yellow for fronto-parietal, green for cingulo-opercular and blue for sensorimotor network.
(DOC)

Figure S2 Illustration of the significant and reliable functional connections with GSR in the young group (**a**) and in the old group (**b**). The young group showed significantly higher test-retest reliability in RSFC than the old group with GSR (Fisher’s exact test: *p*-value = 0.032).
(DOC)

Figure S3 Illustration of the significant and reliable functional connections without GSR in the young group (**a**) and in the old group (**b**). The young group showed significantly higher test-retest reliability in RSFC than the old group without GSR (*p*-value <0.001).
(DOC)

Figure S4 Box plots of multi-scan ICCs for significant and non-significant correlations with GSR (left), and without GSR (right) for young (a) versus old group (b), and for positive significant and negative significant correlations with GSR (left), and without GSR (right) for young (c) versus old group (d). Red lines represent the mean ICCs for those correlations.
(DOC)

Figure S5 Group-averaged multi-scan correlation coefficients plotted against their corresponding multi-scan ICCs with GSR (left) and without GSR (right) for the young group (a) and for the old group (b). Rug plots are shown on each axis representing the distribution of correlations and multi-scan ICCs. Blue dots are multi-scan ICCs and the red lines represent the linear fitting.
(DOC)

Figure S6 Stability within subjects across scans. Shown are box plots of Kendall’s W for all, significant, non-significant, positive significant and negative significant correlations with GSR (left), and without GSR (right) for the young (a) versus the old group (b). Red lines represent the mean values of Kendall’s *W* within subjects across scans.
(DOC)

Figure S7 Stability between-subject within scans. Shown are box plots of Kendall’s *W* for all, significant, non-significant, positive significant and negative significant correlations with GSR (left), and without GSR (right) for the young (a) versus the old group (b). Red lines represent the mean values of Kendall’s *W* between-subject within scans.
(DOC)

Table S1 Listed are 92 ROIs with their MNI coordinates and respective functional networks. 19 ROIs are from the default-mode network, 23 ROIs from the cingulo-opercular network, 17 ROIs from the fronto-parietal network and 33 ROIs from the sensorimotor network.
(DOC)

Table S2 Listed are significant and reliable correlations (i.e., *p*-value <0.05 adjusted by FDR correction, ICC >0.5) with GSR for young group (**a**) and old group (**b**) (only multi-scan ICCs >0.6 are shown here due to the large number of correlations with ICC exceeding 0.5). Mean Rs are group-averaged correlation values from each scan.
(DOC)

Table S3 Listed are significant and reliable correlations (i.e., *p*-value <0.05 adjusted by FDR correction, ICC >0.5) without GSR for young group (**a**) and old group (**b**) (only multi-scan ICCs >0.6 are shown here due to the large number of correlations with ICC exceeding 0.5). Mean Rs are group-averaged correlation values from each scan.
(DOC)
