# Supplementary figures and images for: Age-Related Differences in Test-Retest Reliability in Resting-State Brain Functional Connectivity
Source: PLoS One. 2012 Dec 5;7(12):e49847. doi: 10.1371/journal.pone.0049847 (PMC3515585; doi:10.1371/journal.pone.0049847)

**Figure S1:** Shown are92 regions of interest (ROIs) used in this study taken from Dosenbach et al. (2010).

**
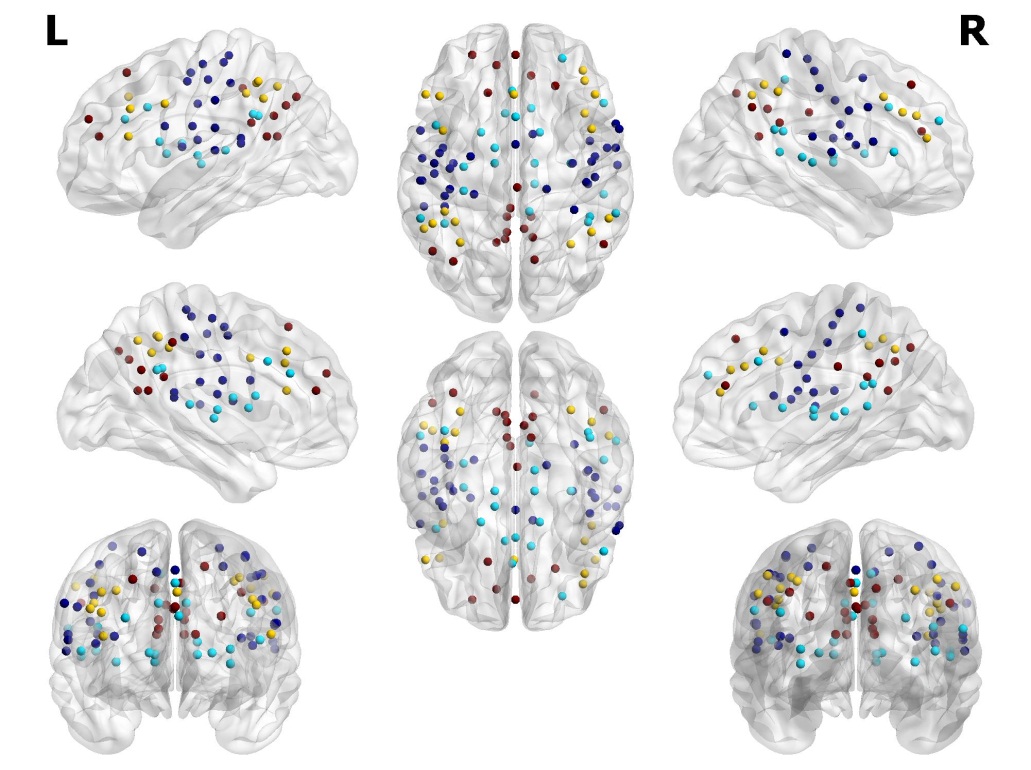
**

Supplement: Figure S1 — Shown are 92 regions of interest (ROIs) used in this study taken from Dosenbach et al. (2010). All 92 ROIs are displayed on a surface rendering of the brain (ICBM 152) visualized with the BrainNet Viewer (http://www.nitrc.org/projects/bnv/). Red dots represent the ROIs from the default mode network, yellow for fronto-parietal, green for cingulo-opercular and blue for sensorimotor network. (DOC) [file pone.0049847.s001.doc]
