# Supplementary material for: Age-Related Differences in Test-Retest Reliability in Resting-State Brain Functional Connectivity
Source: PLoS One. 2012 Dec 5;7(12):e49847. doi: 10.1371/journal.pone.0049847 (PMC3515585; doi:10.1371/journal.pone.0049847)

**Figure S2:** Illustration of the significant and reliable functional connections with GSR in the young group (**a**) and in the old group (**b**).

**a)**


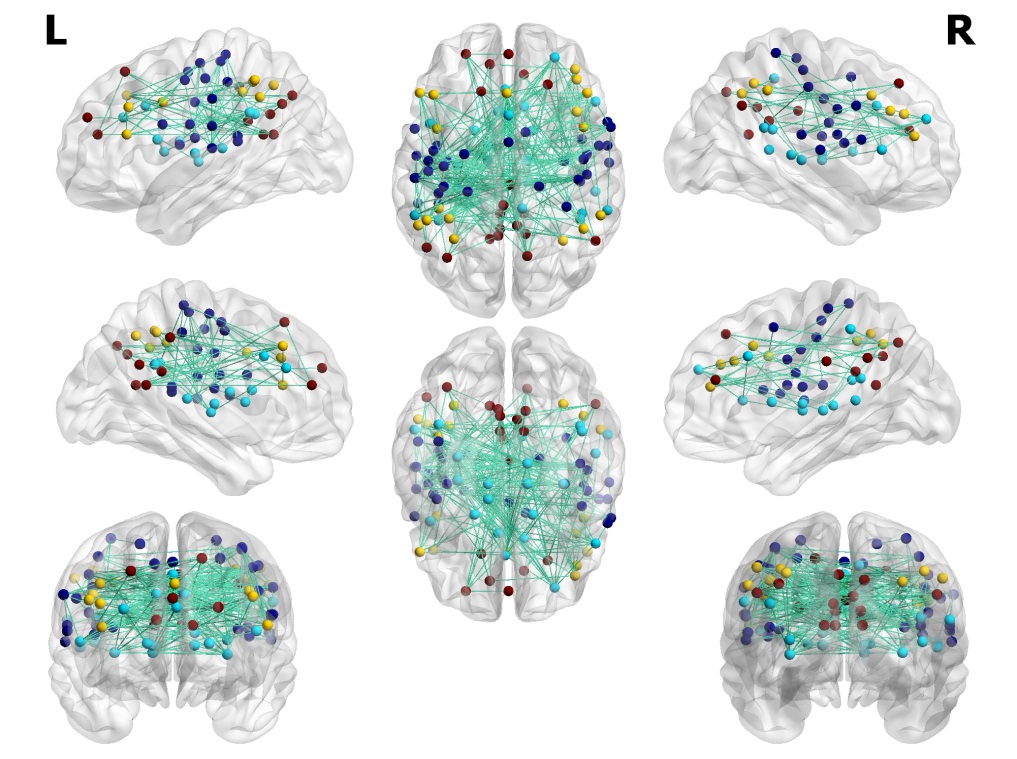


**b)**


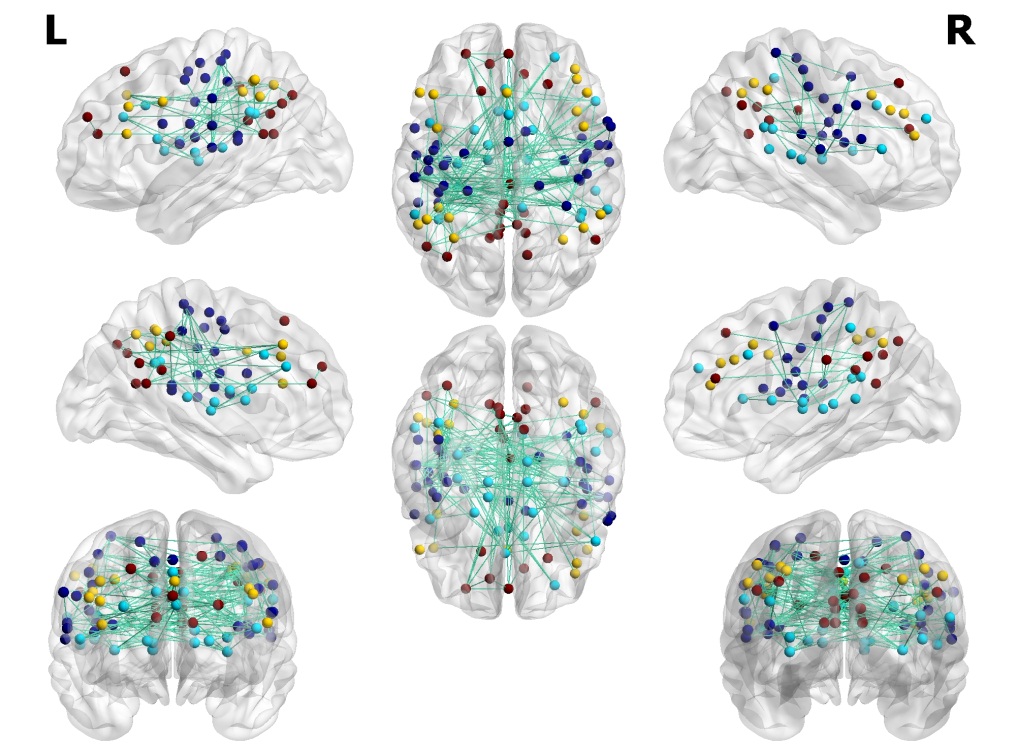

Supplement: Figure S2 — Illustration of the significant and reliable functional connections with GSR in the young group (a) and in the old group (b). The young group showed significantly higher test-retest reliability in RSFC than the old group with GSR (Fisher’s exact test: p-value = 0.032). (DOC) [file pone.0049847.s002.doc]
