# Supplementary material for: Age-Related Differences in Test-Retest Reliability in Resting-State Brain Functional Connectivity
Source: PLoS One. 2012 Dec 5;7(12):e49847. doi: 10.1371/journal.pone.0049847 (PMC3515585; doi:10.1371/journal.pone.0049847)

**Figure S3:** Illustration of the significant and reliable functional connections without GSR in the young group (**a**) and in the old group (**b**).

**a)**


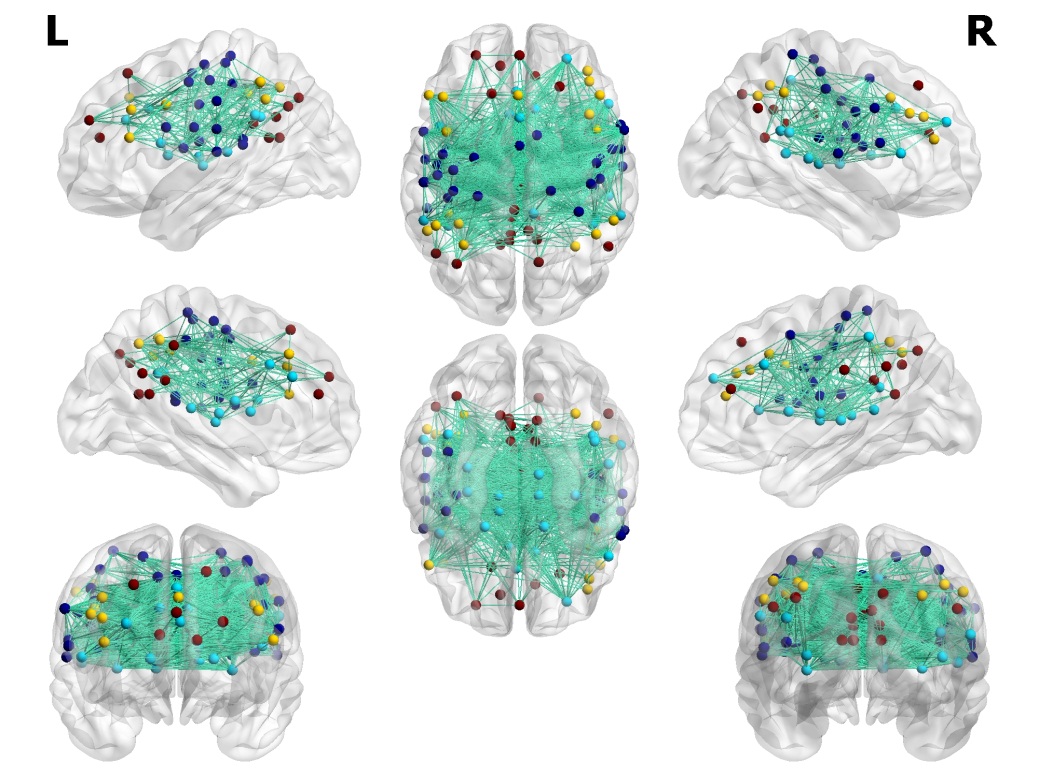


**b)**


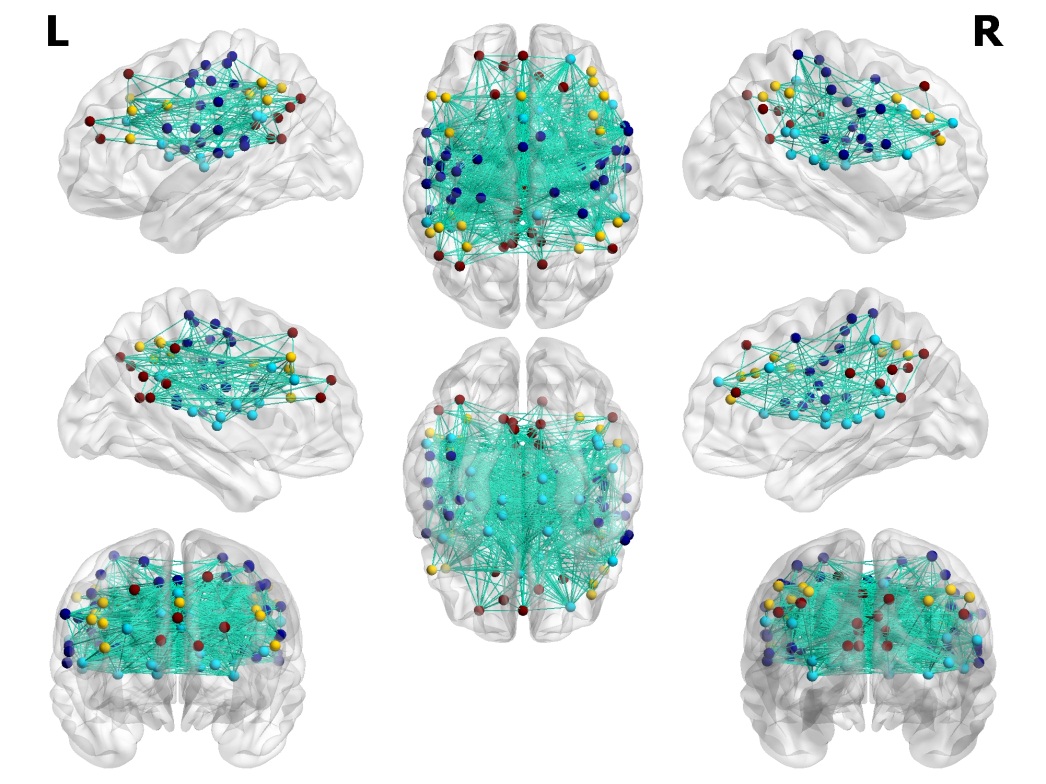

Supplement: Figure S3 — Illustration of the significant and reliable functional connections without GSR in the young group (a) and in the old group (b). The young group showed significantly higher test-retest reliability in RSFC than the old group without GSR (p-value <0.001). (DOC) [file pone.0049847.s003.doc]
