# Supplementary material for: Age-Related Differences in Test-Retest Reliability in Resting-State Brain Functional Connectivity
Source: PLoS One. 2012 Dec 5;7(12):e49847. doi: 10.1371/journal.pone.0049847 (PMC3515585; doi:10.1371/journal.pone.0049847)
Supplement: Figure S4 — Box plots of multi-scan ICCs for significant and non-significant correlations with GSR (left), and without GSR (right) for young (a) versus old group (b), and for positive significant and negative significant correlations with GSR (left), and without GSR (right) for young (c) versus old group (d). Red lines represent the mean ICCs for those correlations. (DOC) [file pone.0049847.s004.doc]

**Figure S4:** Box plots of multi-scan ICCs for significant, non-significant, positive and negative correlations for both groups.

**a)**

**b)**

**c)**

**d)**
