# Supplementary material for: Age-Related Differences in Test-Retest Reliability in Resting-State Brain Functional Connectivity
Source: PLoS One. 2012 Dec 5;7(12):e49847. doi: 10.1371/journal.pone.0049847 (PMC3515585; doi:10.1371/journal.pone.0049847)
Supplement: Figure S5 — Group-averaged multi-scan correlation coefficients plotted against their corresponding multi-scan ICCs with GSR (left) and without GSR (right) for the young group (a) and for the old group (b). Rug plots are shown on each axis representing the distribution of correlations and multi-scan ICCs. Blue dots are multi-scan ICCs and the red lines represent the linear fitting. (DOC) [file pone.0049847.s005.doc]

**Figure S5:** Group-averaged multi-scan correlation coefficients plotted against their corresponding multi-scan ICCs.

**a)**

**b)**
