# Supplementary material for: Age-Related Differences in Test-Retest Reliability in Resting-State Brain Functional Connectivity
Source: PLoS One. 2012 Dec 5;7(12):e49847. doi: 10.1371/journal.pone.0049847 (PMC3515585; doi:10.1371/journal.pone.0049847)
Supplement: Figure S7 — Stability between-subject within scans. Shown are box plots of Kendall’s W for all, significant, non-significant, positive significant and negative significant correlations with GSR (left), and without GSR (right) for the young (a) versus the old group (b). Red lines represent the mean values of Kendall’s W between-subject within scans. (DOC) [file pone.0049847.s007.doc]

**Figure S7:** Stability between-subject within scans.

**a)**

**b)**
