# Supplementary material for: Age-Related Differences in Test-Retest Reliability in Resting-State Brain Functional Connectivity
Source: PLoS One. 2012 Dec 5;7(12):e49847. doi: 10.1371/journal.pone.0049847 (PMC3515585; doi:10.1371/journal.pone.0049847)
Supplement: Table S1 — Listed are 92 ROIs with their MNI coordinates and respective functional networks. 19 ROIs are from the default-mode network, 23 ROIs from the cingulo-opercular network, 17 ROIs from the fronto-parietal network and 33 ROIs from the sensorimotor network. (DOC) [file pone.0049847.s008.doc]

**Table S1: Listed are 92 ROIs with their MNI coordinates and respective functional networks.**

| **x** | **y** | **z** | **ROI lable** | **Network** |
| --- | --- | --- | --- | --- |
| 0 | 51 | 32 | M_mPFC | default |
| -25 | 51 | 27 | L_aPFC_2 | default |
| 27 | 49 | 26 | R_aPFC_2 | cingulo-opercular |
| -11 | 45 | 17 | L_vmPFC | default |
| 39 | 42 | 16 | R_vlPFC | fronto-parietal |
| 9 | 39 | 20 | R_ACC | default |
| 40 | 36 | 29 | R_dlPFC_1 | fronto-parietal |
| 23 | 33 | 47 | R_sup_frontal | default |
| -2 | 30 | 27 | M_ACC_1 | cingulo-opercular |
| -16 | 29 | 54 | L_sup_frontal | default |
| -1 | 28 | 40 | M_ACC_2 | fronto-parietal |
| 46 | 28 | 31 | R_dlPFC_2 | fronto-parietal |
| -52 | 28 | 17 | L_vPFC | fronto-parietal |
| -44 | 27 | 33 | L_dlPFC | fronto-parietal |
| 51 | 23 | 8 | R_vFC_1 | cingulo-opercular |
| 9 | 20 | 34 | R_dACC | cingulo-opercular |
| 40 | 17 | 40 | R_dFC_1 | fronto-parietal |
| -6 | 17 | 34 | L_basal_ganglia_1 | cingulo-opercular |
| 0 | 15 | 45 | M_mFC | cingulo-opercular |
| 58 | 11 | 14 | R_frontal_1 | sensorimotor |
| -46 | 10 | 14 | L_vFC_1 | cingulo-opercular |
| 44 | 8 | 34 | R_dFC_2 | fronto-parietal |
| 60 | 8 | 34 | R_dFC_3 | sensorimotor |
| -42 | 7 | 36 | L_dFC | fronto-parietal |
| -55 | 7 | 23 | L_vFC_2 | sensorimotor |
| -20 | 6 | 7 | L_basal_ganglia_2 | cingulo-opercular |
| 14 | 6 | 7 | R_basal_ganglia_1 | cingulo-opercular |
| 10 | 5 | 51 | R_pre_SMA | sensorimotor |
| 43 | 1 | 12 | R_vFC_2 | sensorimotor |
| 0 | -1 | 52 | M_SMA | sensorimotor |
| 53 | -3 | 32 | R_frontal_2 | sensorimotor |
| 58 | -3 | 17 | R_precentral_gyrus_1 | sensorimotor |
| -12 | -3 | 13 | L_thalamus_1 | cingulo-opercular |
| -42 | -3 | 11 | L_mid_insula_1 | sensorimotor |
| -44 | -6 | 49 | L_precentral_gyrus_1 | sensorimotor |
| -26 | -8 | 54 | L_parietal_1 | sensorimotor |
| 46 | -8 | 24 | R_precentral_gyrus_2 | sensorimotor |
| -54 | -9 | 23 | L_precentral_gyrus_2 | sensorimotor |
| 44 | -11 | 38 | R_precentral_gyrus_3 | sensorimotor |
| -47 | -12 | 36 | L_parietal_2 | sensorimotor |
| 33 | -12 | 16 | R_mid_insula_1 | sensorimotor |
| -36 | -12 | 15 | L_mid_insula_2 | sensorimotor |
| -12 | -12 | 6 | L_thalamus_2 | cingulo-opercular |
| 11 | -12 | 6 | R_thalamus_1 | cingulo-opercular |
| 32 | -12 | 2 | R_mid_insula_2 | cingulo-opercular |
| 59 | -13 | 8 | R_temporal_1 | sensorimotor |
| -30 | -14 | 1 | L_mid_insula_3 | cingulo-opercular |
| -38 | -15 | 59 | L_parietal_3 | sensorimotor |
| -47 | -18 | 50 | L_parietal_4 | sensorimotor |
| 46 | -20 | 45 | R_parietal_1 | sensorimotor |
| -55 | -22 | 38 | L_parietal_5 | sensorimotor |
| -54 | -22 | 22 | L_precentral_gyrus_3 | sensorimotor |
| -54 | -22 | 9 | L_temporal_1 | sensorimotor |
| 41 | -23 | 55 | R_parietal_2 | sensorimotor |
| 42 | -24 | 17 | R_post_insula | sensorimotor |
| 11 | -24 | 2 | R_basal_ganglia_2 | cingulo-opercular |
| 1 | -26 | 31 | M_post_cingulate | default |
| 18 | -27 | 62 | R_parietal_3 | sensorimotor |
| -38 | -27 | 60 | L_parietal_6 | sensorimotor |
| -30 | -28 | 9 | L_post_insula | cingulo-opercular |
| -24 | -30 | 64 | L_parietal_7 | sensorimotor |
| 51 | -30 | 5 | R_temporal_2 | cingulo-opercular |
| -41 | -31 | 48 | L_post_parietal_1 | sensorimotor |
| -41 | -37 | 16 | L_temporal_2 | sensorimotor |
| -53 | -37 | 13 | L_temporal_3 | sensorimotor |
| -3 | -38 | 45 | L_precuneus_1 | default |
| 34 | -39 | 65 | R_sup_parietal | sensorimotor |
| 8 | -40 | 50 | R_precuneus_1 | cingulo-opercular |
| -41 | -40 | 42 | L_IPL_1 | fronto-parietal |
| 58 | -41 | 20 | R_parietal_4 | cingulo-opercular |
| -5 | -43 | 25 | L_post_cingulate_1 | default |
| 9 | -43 | 25 | R_precuneus_2 | default |
| 43 | -43 | 8 | R_temporal_3 | cingulo-opercular |
| 54 | -44 | 43 | R_IPL_1 | fronto-parietal |
| -55 | -44 | 30 | L_parietal_8 | cingulo-opercular |
| -35 | -46 | 48 | L_post_parietal_2 | fronto-parietal |
| 42 | -46 | 21 | R_sup_temporal | cingulo-opercular |
| -48 | -47 | 49 | L_IPL_2 | fronto-parietal |
| -41 | -47 | 29 | L_angular_gyrus_1 | cingulo-opercular |
| -53 | -50 | 39 | L_IPL_3 | fronto-parietal |
| 5 | -50 | 33 | R_precuneus_3 | default |
| 44 | -52 | 47 | R_IPL_2 | fronto-parietal |
| -5 | -52 | 17 | L_post_cingulate_2 | default |
| 10 | -55 | 17 | R_post_cingulate | default |
| -6 | -56 | 29 | L_precuneus_2 | default |
| -32 | -58 | 46 | L_IPS_1 | fronto-parietal |
| -11 | -58 | 17 | L_post_cingulate_3 | default |
| 32 | -59 | 41 | R_IPS | fronto-parietal |
| 51 | -59 | 34 | R_angular_gyrus | default |
| -48 | -63 | 35 | L_angular_gyrus_2 | default |
| 11 | -68 | 42 | R_precuneus_4 | default |
| -36 | -69 | 40 | L_IPS_2 | default |
