# Supplementary material for: Age-Related Differences in Test-Retest Reliability in Resting-State Brain Functional Connectivity
Source: PLoS One. 2012 Dec 5;7(12):e49847. doi: 10.1371/journal.pone.0049847 (PMC3515585; doi:10.1371/journal.pone.0049847)
Supplement: Table S2 — Listed are significant and reliable correlations (i.e., p-value <0.05 adjusted by FDR correction, ICC >0.5) with GSR for young group (a) and old group (b) (only multi-scan ICCs >0.6 are shown here due to the large number of correlations with ICC exceeding 0.5). Mean Rs are group-averaged correlation values from each scan. (DOC) [file pone.0049847.s009.doc]

**Table S2** Listed are significant and reliable correlations (i.e., *p*-value < 0.05 adjusted by FDR correction, ICC > 0.5) with GSR for both groups.

**a)**

| **Young Group** | | | | | | | | |
| --- | --- | --- | --- | --- | --- | --- | --- | --- |
| **Correlation** | **ICC** | **Mean R scan 1** | | **Mean R scan 2** | | **Mean R scan 3** | | **Networks** |
| R_thalamus_1 - L_thalamus_2 | 0.78 | | 0.63 | 0.61 | 0.61 | | cingulo-opercular - cingulo-opercular | |
| R_IPL_2 - R_dFC_2 | 0.77 | | 0.30 | 0.23 | 0.23 | | fronto-parietal - fronto-parietal | |
| M_ACC_2 - M_ACC_1 | 0.75 | | 0.52 | 0.48 | 0.43 | | fronto-parietal - cingulo-opercular | |
| L_precuneus_2 - L_post_cingulate_2 | 0.72 | | 0.71 | 0.72 | 0.69 | | default - default | |
| L_precuneus_2 - L_IPL_3 | 0.70 | | 0.16 | 0.18 | 0.17 | | default - fronto-parietal | |
| L_mid_insula_2 - R_precentral_gyrus_2 | 0.70 | | 0.33 | 0.29 | 0.35 | | sensorimotor - sensorimotor | |
| L_post_cingulate_3 - L_post_cingulate_2 | 0.70 | | 0.71 | 0.70 | 0.72 | | default - default | |
| R_angular_gyrus - L_precuneus_1 | 0.70 | | 0.25 | 0.19 | 0.18 | | default - default | |
| L_post_cingulate_3 - L_precuneus_2 | 0.70 | | 0.62 | 0.61 | 0.59 | | default - default | |
| L_precuneus_2 - R_IPL_1 | 0.68 | | -0.17 | -0.18 | -0.17 | | default - fronto-parietal | |
| L_IPL_2 - L_sup_frontal | 0.67 | | 0.15 | 0.16 | 0.16 | | fronto-parietal - default | |
| L_precuneus_2 - L_post_cingulate_1 | 0.66 | | 0.54 | 0.62 | 0.58 | | default - default | |
| L_precentral_gyrus_3 - L_mid_insula_2 | 0.66 | | 0.40 | 0.43 | 0.42 | | sensorimotor - sensorimotor | |
| L_post_cingulate_1 - M_post_cingulate | 0.66 | | 0.60 | 0.61 | 0.62 | | default - default | |
| R_precentral_gyrus_3 - L_precentral_gyrus_2 | 0.66 | | 0.50 | 0.45 | 0.45 | | sensorimotor - sensorimotor | |
| L_precentral_gyrus_3 - R_mid_insula_1 | 0.66 | | 0.36 | 0.35 | 0.29 | | sensorimotor - sensorimotor | |
| L_precentral_gyrus_3 - R_precentral_gyrus_2 | 0.65 | | 0.29 | 0.32 | 0.33 | | sensorimotor - sensorimotor | |
| L_angular_gyrus_2 - L_IPL_2 | 0.65 | | 0.17 | 0.23 | 0.23 | | default - fronto-parietal | |
| R_IPL_1 - R_dlPFC_1 | 0.65 | | 0.39 | 0.39 | 0.45 | | fronto-parietal - fronto-parietal | |
| L_parietal_5 - L_precentral_gyrus_2 | 0.65 | | 0.27 | 0.34 | 0.37 | | sensorimotor - sensorimotor | |
| R_IPL_1 - M_mFC | 0.65 | | 0.18 | 0.22 | 0.21 | | fronto-parietal - cingulo-opercular | |
| L_IPL_3 - L_sup_frontal | 0.64 | | 0.31 | 0.34 | 0.33 | | fronto-parietal - default | |
| R_angular_gyrus - L_IPL_3 | 0.64 | | 0.32 | 0.38 | 0.37 | | default - fronto-parietal | |
| L_parietal_4 - R_frontal_2 | 0.64 | | 0.25 | 0.31 | 0.35 | | sensorimotor - sensorimotor | |
| L_IPL_2 - L_parietal_8 | 0.64 | | 0.17 | 0.22 | 0.22 | | fronto-parietal - cingulo-opercular | |
| R_dFC_2 - L_basal_ganglia_1 | 0.63 | | 0.14 | 0.16 | 0.15 | | fronto-parietal - cingulo-opercular | |
| L_precuneus_1 - M_post_cingulate | 0.63 | | 0.32 | 0.33 | 0.36 | | default - default | |
| L_parietal_8 - L_dFC | 0.63 | | 0.14 | 0.13 | 0.13 | | cingulo-opercular - fronto-parietal | |
| R_IPL_1 - R_vlPFC | 0.63 | | 0.33 | 0.37 | 0.34 | | fronto-parietal - fronto-parietal | |
| R_frontal_2 - R_dFC_2 | 0.63 | | 0.18 | 0.21 | 0.22 | | sensorimotor - fronto-parietal | |
| R_temporal_1 - R_precentral_gyrus_2 | 0.63 | | 0.28 | 0.28 | 0.30 | | sensorimotor - sensorimotor | |
| L_IPL_3 - L_aPFC_2 | 0.63 | | 0.21 | 0.15 | 0.18 | | fronto-parietal - default | |
| L_parietal_2 - R_precentral_gyrus_1 | 0.63 | | 0.48 | 0.46 | 0.44 | | sensorimotor - sensorimotor | |
| R_IPL_2 - R_dlPFC_2 | 0.63 | | 0.45 | 0.44 | 0.41 | | fronto-parietal - fronto-parietal | |
| L_IPL_2 - R_IPL_1 | 0.62 | | 0.32 | 0.38 | 0.42 | | fronto-parietal - fronto-parietal | |
| R_IPL_2 - R_dlPFC_1 | 0.62 | | 0.32 | 0.34 | 0.38 | | fronto-parietal - fronto-parietal | |
| R_precentral_gyrus_3 - R_frontal_2 | 0.61 | | 0.60 | 0.56 | 0.58 | | sensorimotor - sensorimotor | |
| L_aPFC_2 - M_mPFC | 0.61 | | 0.29 | 0.23 | 0.32 | | default - default | |
| R_precuneus_3 - R_precuneus_2 | 0.61 | | 0.56 | 0.57 | 0.52 | | default - default | |
| L_IPL_3 - L_post_cingulate_1 | 0.61 | | 0.12 | 0.15 | 0.14 | | fronto-parietal - default | |
| L_post_cingulate_1 - L_vmPFC | 0.61 | | 0.20 | 0.22 | 0.29 | | default - default | |
| L_IPS_2 - L_IPS_1 | 0.61 | | 0.28 | 0.36 | 0.41 | | default - fronto-parietal | |
| L_parietal_4 - R_precentral_gyrus_3 | 0.61 | | 0.34 | 0.39 | 0.39 | | sensorimotor - sensorimotor | |
| L_dlPFC - R_dlPFC_1 | 0.61 | | 0.36 | 0.31 | 0.31 | | fronto-parietal - fronto-parietal | |
| L_IPL_3 - R_sup_frontal | 0.61 | | 0.18 | 0.20 | 0.15 | | fronto-parietal - default | |
| L_IPL_2 - L_dFC | 0.61 | | 0.32 | 0.36 | 0.24 | | fronto-parietal - fronto-parietal | |
| M_SMA - R_dFC_3 | 0.61 | | 0.23 | 0.24 | 0.19 | | sensorimotor - sensorimotor | |
| R_angular_gyrus - R_dFC_3 | 0.61 | | -0.19 | -0.19 | -0.20 | | default - sensorimotor | |
| L_IPL_3 - L_parietal_8 | 0.60 | | 0.39 | 0.43 | 0.46 | | fronto-parietal - cingulo-opercular | |
| L_parietal_7 - R_parietal_1 | 0.60 | | 0.23 | 0.30 | 0.32 | | sensorimotor - sensorimotor | |
| L_vFC_2 - L_vPFC | 0.60 | | 0.32 | 0.32 | 0.33 | | sensorimotor - fronto-parietal | |
| R_IPL_2 - R_dFC_1 | 0.60 | | 0.41 | 0.39 | 0.42 | | fronto-parietal - fronto-parietal | |
| L_post_cingulate_2 - R_precuneus_3 | 0.60 | | 0.55 | 0.56 | 0.54 | | default - default | |
| L_post_cingulate_3 - L_post_cingulate_1 | 0.60 | | 0.37 | 0.43 | 0.40 | | default - default | |
| R_IPL_2 - R_vlPFC | 0.60 | | 0.31 | 0.34 | 0.30 | | fronto-parietal - fronto-parietal | |

**b)**

| **Old Group** | | | | | | | | |
| --- | --- | --- | --- | --- | --- | --- | --- | --- |
| **Correlation** | **ICC** | **Mean R**  **scan 1** | | **Mean R**  **scan 2** | | **Mean R**  **scan 3** | | **Networks** |
| R_angular_gyrus - L_post_cingulate_3 | 0.79 | | 0.35 | 0.24 | 0.31 | | default - default | |
| R_angular_gyrus - R_sup_frontal | 0.73 | | 0.39 | 0.33 | 0.34 | | default - default | |
| R_dFC_2 - R_dlPFC_1 | 0.72 | | 0.27 | 0.26 | 0.30 | | fronto-parietal - fronto-parietal | |
| R_angular_gyrus - R_post_cingulate | 0.72 | | 0.43 | 0.32 | 0.37 | | default - default | |
| R_angular_gyrus - L_post_cingulate_2 | 0.71 | | 0.41 | 0.33 | 0.36 | | default - default | |
| L_parietal_4 - L_parietal_3 | 0.70 | | 0.59 | 0.55 | 0.58 | | sensorimotor - sensorimotor | |
| L_parietal_3 - R_precentral_gyrus_3 | 0.68 | | 0.17 | 0.29 | 0.21 | | sensorimotor - sensorimotor | |
| R_dFC_2 - M_ACC_2 | 0.68 | | 0.27 | 0.21 | 0.28 | | fronto-parietal - fronto-parietal | |
| L_IPS_1 - L_post_parietal_2 | 0.68 | | 0.52 | 0.53 | 0.53 | | fronto-parietal - fronto-parietal | |
| R_dlPFC_2 - R_aPFC_2 | 0.68 | | 0.26 | 0.28 | 0.23 | | fronto-parietal - cingulo-opercular | |
| R_dlPFC_1 - L_aPFC_2 | 0.67 | | 0.24 | 0.35 | 0.24 | | fronto-parietal - default | |
| R_dFC_2 - R_dlPFC_2 | 0.67 | | 0.40 | 0.40 | 0.41 | | fronto-parietal - fronto-parietal | |
| R_IPL_2 - L_post_parietal_2 | 0.66 | | 0.17 | 0.22 | 0.22 | | fronto-parietal - fronto-parietal | |
| L_parietal_8 - R_parietal_4 | 0.66 | | 0.29 | 0.24 | 0.35 | | cingulo-opercular - cingulo-opercular | |
| R_precentral_gyrus_3 - L_vFC_2 | 0.66 | | 0.32 | 0.25 | 0.28 | | sensorimotor - sensorimotor | |
| L_aPFC_2 - M_mPFC | 0.65 | | 0.41 | 0.39 | 0.39 | | default - default | |
| L_precuneus_2 - R_post_cingulate | 0.65 | | 0.64 | 0.58 | 0.63 | | default - default | |
| M_ACC_1 - R_dlPFC_1 | 0.65 | | 0.30 | 0.32 | 0.33 | | cingulo-opercular - fronto-parietal | |
| L_parietal_6 - L_parietal_4 | 0.65 | | 0.62 | 0.62 | 0.59 | | sensorimotor - sensorimotor | |
| L_angular_gyrus_2 - R_sup_frontal | 0.65 | | 0.23 | 0.21 | 0.22 | | default - default | |
| L_precentral_gyrus_1 - L_vFC_2 | 0.65 | | 0.13 | 0.19 | 0.21 | | sensorimotor - sensorimotor | |
| L_angular_gyrus_2 - L_sup_frontal | 0.65 | | 0.39 | 0.29 | 0.31 | | default - default | |
| R_precuneus_3 - R_sup_temporal | 0.65 | | 0.22 | 0.19 | 0.20 | | default - cingulo-opercular | |
| R_post_cingulate - R_sup_frontal | 0.64 | | 0.29 | 0.21 | 0.22 | | default - default | |
| L_post_cingulate_2 - R_precuneus_2 | 0.64 | | 0.48 | 0.45 | 0.52 | | default - default | |
| R_IPL_2 - L_IPL_1 | 0.63 | | 0.23 | 0.21 | 0.23 | | fronto-parietal - fronto-parietal | |
| M_ACC_2 - L_aPFC_2 | 0.63 | | 0.32 | 0.32 | 0.26 | | fronto-parietal - default | |
| R_dFC_1 - M_ACC_2 | 0.63 | | 0.51 | 0.41 | 0.45 | | fronto-parietal - fronto-parietal | |
| R_precuneus_3 - R_sup_frontal | 0.63 | | 0.36 | 0.30 | 0.28 | | default - default | |
| L_post_cingulate_2 - R_precuneus_3 | 0.63 | | 0.55 | 0.54 | 0.51 | | default - default | |
| R_dFC_2 - R_dFC_1 | 0.62 | | 0.32 | 0.33 | 0.37 | | fronto-parietal - fronto-parietal | |
| L_sup_frontal - L_vmPFC | 0.62 | | 0.21 | 0.16 | 0.25 | | default - default | |
| L_parietal_6 - L_precentral_gyrus_2 | 0.62 | | 0.18 | 0.16 | 0.21 | | sensorimotor - sensorimotor | |
| R_post_cingulate - R_precuneus_3 | 0.62 | | 0.59 | 0.52 | 0.55 | | default - default | |
| L_angular_gyrus_2 - L_post_cingulate_3 | 0.62 | | 0.30 | 0.24 | 0.24 | | default - default | |
| R_aPFC_2 - L_aPFC_2 | 0.62 | | 0.55 | 0.56 | 0.52 | | cingulo-opercular - default | |
| L_post_cingulate_3 - R_sup_frontal | 0.61 | | 0.28 | 0.22 | 0.18 | | default - default | |
| L_precuneus_2 - L_post_cingulate_2 | 0.61 | | 0.70 | 0.66 | 0.66 | | default - default | |
| L_parietal_6 - L_parietal_2 | 0.61 | | 0.23 | 0.18 | 0.22 | | sensorimotor - sensorimotor | |
| L_IPS_2 - L_post_cingulate_2 | 0.61 | | 0.36 | 0.33 | 0.30 | | default - default | |
| L_precuneus_1 - L_parietal_5 | 0.60 | | 0.16 | 0.14 | 0.25 | | default - sensorimotor | |
| M_ACC_2 - M_ACC_1 | 0.60 | | 0.49 | 0.47 | 0.45 | | fronto-parietal - cingulo-opercular | |
| L_parietal_4 - L_precentral_gyrus_1 | 0.60 | | 0.47 | 0.46 | 0.51 | | sensorimotor - sensorimotor | |
| R_angular_gyrus - R_IPS | 0.60 | | 0.19 | 0.25 | 0.18 | | default - fronto-parietal | |
